# A Competing Hydrogen Bond Network Offers Access to a New Conformation in 24-Atom Triazine Macrocycles

**DOI:** 10.3390/molecules30224475

**Published:** 2025-11-20

**Authors:** K. Harsha Vardan Reddy, Arshad Mehmood, Akop Yepremyan, Eric E. Simanek

**Affiliations:** 1 Department of Chemistry & Biochemistry, Texas Christian University, Fort Worth, TX 76129, USA; h.r.kasireddy@tcu.edu (K.H.V.R.);; 2 Institute for Advanced Computational Science, Stony Brook University, Stony Brook, NY 11794, USA

**Keywords:** macrocycle, triazine, rotamer, conformation, structure determination, isomer, intramolecular hydrogen bond network, variable temperature NMR, conformer, QTAIM

## Abstract

For a family of 24-atom triazine macrocycles, a single intramolecular hydrogen bond (IMHB) network leads to a conserved, hinge-like motif in solution. Modifications to the backbone of these macrocycles preserve this motif. Modifications to peripheral sites lead to conformational isomers due to hindered bond rotation while conserving the hinge motif. Here, a competitive IMHB network is introduced by the addition of a hydrogen bond donor on the periphery. Cyclization remains quantitative, but multiple conformers result. Three conformers are derived from the hinge motif. Three others are attributed to a new motif that utilizes the new IMHB network. Crystallographic analysis confirms this hypothesis and establishes that this new motif differs significantly from the original with respect to overall shape and disposition of groups. Variable temperature ^1^H NMR spectroscopy is used to partially assign the spectra because conformers adopting the hinge motif undergo dynamic motion on the NMR timescale, while the new motif appears static. QTAIM analysis corroborates the hydrogen bond designations in the new conformer and categorizes these interactions as moderate and strong.

## 1. Introduction

Interest in macrocycles derived from dynamic covalent chemistry stems from potential applications in medicine [1,2,3] and materials science [4,5,6,7,8,9] as well as their utility in studying molecular behavior, including physical properties, sorting, and other supra- and macromolecular phenomena [10,11,12,13,14].

Our interest in macrocycles was sparked with the serendipitous observation that self-reactive monomers bearing hydrazine and acetal groups undergo dimerization in the presence of acid in quantitative yields [15]. The scope of this reaction has been probed as a function of ring size [16], as well as the alkylation state of the hydrazine [17], the choice of amino acid [18], and/or substitution at the auxiliary site on the triazine ring [19]. NMR and crystallographic studies have revealed that regardless of derivatization to date, these molecules adopt a common, hinge-like motif and display dynamic hinge-like motion at room temperature on the NMR timescale [20,21,22]. These shared elements (as well as the quantitative yields) are attributed to an intramolecular hydrogen bond (IMHB) network [23].

**Figure 1:**
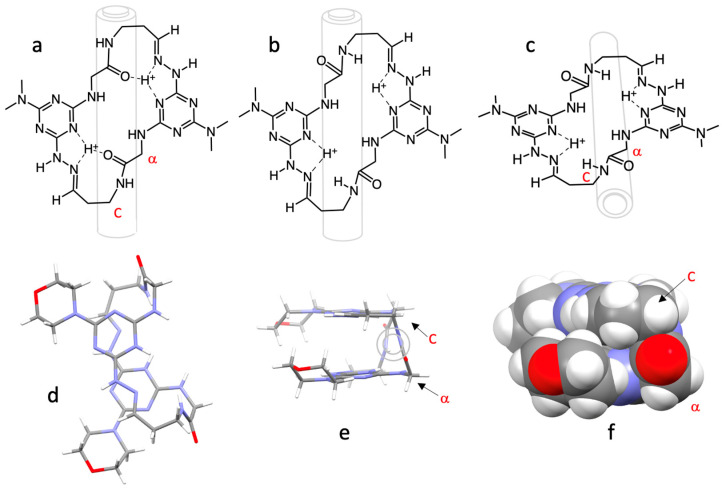
**Glycine macrocycles with different substituents illustrating the hinge motif and hinge axis.** See text for additional details. Panels (**a**–**c**) overlay the hinge on the structure and indicate reporting methylene groups (a and C). Panels (**d**–**f**) derive from crystal structure data of the morpholine derivative.

The closed hinge motif is illustrated in Figure 1. Panels a and b depict the macrocycles and indicate the reporters (α and C) when the carbonyl is engaged in the IMHB (a) and when it is not (b). Panel c shows the open hinge from an edge-on view. For panels a–c, the glycine macrocycle is shown with dimethylamine substituents at the auxiliary (exocyclic) position. This macrocycle provides readily interpretable NMR spectra. Panels d–f show the crystal structures obtained when morpholine is incorporated. Crystals of the macrocycle with the dimethyl amine groups have not been obtained. Panel d shows the closed hinge from the top. Panels e and f show the closed hinge from the side, with reporting methylene groups indicated.

The open hinge cannot be observed directly. Hinging allows interconversion between enantiomeric states. Coalescence of resonances corresponding to pseudo-equatorial and pseudo-axial methylene groups on the macrocycle backbone (α and C) is observed using variable temperature NMR spectroscopy.

**Figure 2:**
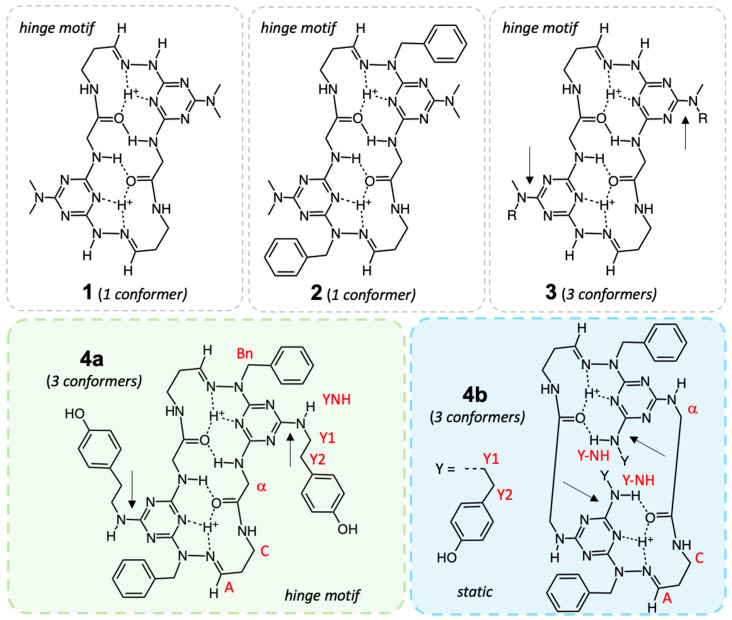
**Four different macrocycles.** The conserved IMHB network in **1**–**3** affords macrocycles displaying a hinge-like motif with hinge motion observable on the NMR timescale. For **1** and **2**, the dimethylamine auxiliary groups (red) provide convenient spectral handles, and one conformer is observed for each in solution. For **3**, the introduction of unsymmetric amines at the auxiliary position (-NMeR) leads to three conformations due to hindered rotation about the triazine—NMeR bond [11]. Here, R is Me, Et, or Bn. These conformers still adopt a hinge-like motif and show hinge motion. For **4**, in addition to the hinge motif (**4a**), a hydrogen bond donor, Y-NH, at the auxiliary position offers an alternative IMHB network and a new, static conformation (**4b**).

Examples of macrocycles that adopt the hinge motif are shown in Figure 2. Hinging has been observed experimentally for **1**–**3**, and the closed hinge is observed in solution by ^1^H NMR spectroscopy. Initially, the auxiliary amine chosen was symmetric to simplify analysis [15,16,17,18,19,20,21,22]. Dimethylamine (used in **1** and **2**) provided readily identified singlets in ^1^H NMR spectra. These singlets are consistent with a single conformation in solution. Morpholine derivatives provided crystalline materials amenable to study by single-crystal X-ray diffraction. The solid-state structures obtained are largely identical to the solution structure. When the auxiliary amine is unsymmetric (Figure 1; **3** R = Et), the hinge motif is conserved, but three conformers are accessible due to hindered rotation about the triazine-NMeR bond (Figure S1, Supplementary Materials). These bonds are indicated with arrows.

Introduction of a primary amine, tyramine, increases structural complexity. Here, we report that **4a** adopts the hinge motif, and hinging is observed experimentally. The introduction of a competitive hydrogen bond donor offers the potential for access to a new IMHB and a structure, **4b**, that is markedly different from those previously observed. Throughout this report, light green is used to indicate elements of the hinge motif and light blue for elements of the new, static motif.

Here, we establish that the introduction of tyramine, a hydrogen bond donor, at the auxiliary position to yield **4,** leads to two different motifs (**4a** and **4b**) differing in the IMHB network. The expected hinge motif (**4a**) is observed, and three conformers can be identified. These conformers undergo hinge-like motion (*vide infra*) on the NMR timescale [20,21,22]. The other motif (**4b**) is reflected in the crystal structure obtained (vide infra) and appears static on the NMR timescale. Recent advances in the interpretation of the ^1^H NMR spectra derived from macrocycles bearing unsymmetric auxiliary groups (**3**) confirm that **4** exists as a mixture of six conformers [24]. Overlap in the ^1^H NMR spectra precludes complete assignment of all resonances, but the presence of dynamic hinge motion in half of these conformers (and static behavior of the others) proves critical in interpreting these results.

## 2. Results

### 2.1. Synthesis

**Scheme 1:**
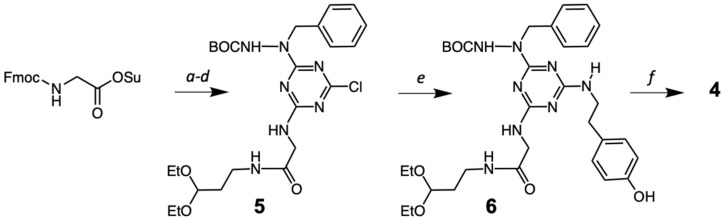
Synthetic route employed, starting with the succinimide (Su) ester of Fmoc-glycine. (a) 1-amino-3,3-diethoxypropane, THF, DIPEA, rt; (b) diethylamine; (c) C_3_N_3_Cl_3_, DIPEA, THF, −10 °C; (d) BnHNNHBOC, THF, DIPEA, rt; (e) tyramine, DIPEA, THF, rt; (f) TFA.

The synthetic route to **4** is shown in Scheme 1. Briefly, a four-step, one-pot procedure is employed to generate intermediate **5**. First, Fmoc-Gly-OSu is reacted with 1,1-diethoxypropan-3-amine. Second, upon completion of the reaction (as monitored with thin-layer chromatography, TLC), diethylamine is added and the reaction is stirred until Fmoc cleavage is established by TLC. Solvent and residual diethylamine are removed using rotary evaporation. Third, the resulting residue is dissolved in THF and added dropwise to an iced solution of cyanuric chloride and diisopropylethylamine (DIPEA) in THF. Fourth, BnHNNHBOC is added to the solution with DIPEA. Upon completion, intermediate **5** is isolated by silica gel chromatography and characterized. To arrive at the monomer, **6**, intermediate **5** is dissolved in THF, and tyramine is added. The monomer is purified by silica gel chromatography. While all steps could be affected in the same pot, we elected to isolate intermediates to reduce the likelihood of impurities due to over- or under-addition to cyanuric chloride. Treating monomer **6** with trifluoroacetic acid yields **4**.

### 2.2. Crystallography

**Figure 3:**
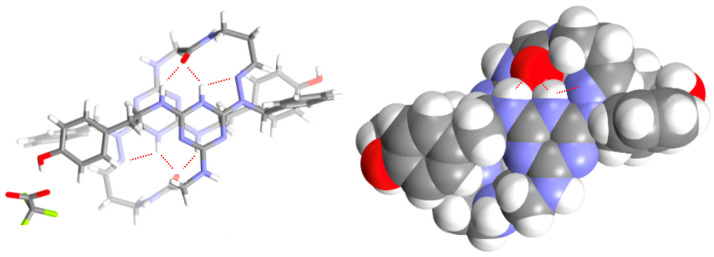
The crystal structure of **4b**. The IMHB networks between the carbonyl of one subunit and the protonated triazine and tyramine-NH are shown with dotted lines. The trifluoroacetate group is omitted for clarity in the space-filling model.

Crystals suitable for X-ray diffraction were obtained by slow evaporation of a solution of **4** in methanol with advantageous water. Figure 3 shows that the structure obtained corresponds to **4b**. The triazine rings are protonated, and the trifluoracetate counterion is not intimately associated.

Other features of interest include the p-p stacking between the subunits, with the triazine ring of one subunit making contact with the hydrazone of the other. This stacking, as well as the geometry of the hydrazone, is conserved across macrocycles **1**–**4**, although here, the orientation of the triazine groups differs.

In addition, the IMHB network can be identified, wherein the carbonyl of one subunit engages in two hydrogen bonds with the other subunit. Both the H^+^ and the tyramine-NH (Y-NH) serve as hydrogen bond donors. The hydrazone contributes to this stabilization [23]. Tables S1 and S2 in the Supplementary Materials provide additional information. This structure is deposited with the Cambridge Crystallographic Data Centre as supplementary publication CCDC 2497285.

### 2.3. NMR Analysis

**Figure 4:**
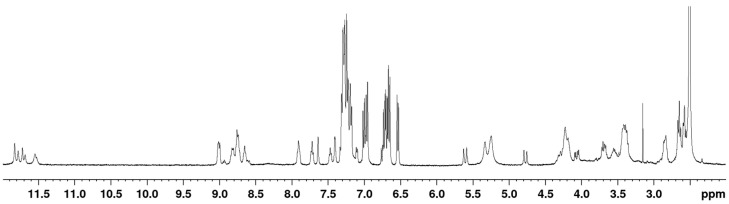
The 400 MHz ^1^H NMR spectrum of **4** in DMSO-*d_6_* with TFA. Broad resonances at 3.4, 4.2, 5.3, and 5.4 are consistent with hinge motion for a subpopulation of these macrocycles (vide infra).

Figure 4 shows the 400 MHz ^1^H NMR spectrum of the reaction residue (**4**) dissolved in DMSO-*d_6_* in the presence of trifluoroacetic acid. Different regions of the spectrum have proven useful for determining the number, conformation, and dynamic behavior of these macrocycles [7,11]. Most notable are the multiple resonances between 11.5 and 12.0 ppm corresponding to H^+^ resonances. Figures S2–S13 of the Supplementary Materials provide 1-D and 2-D NMR spectra.

**Figure 5:**
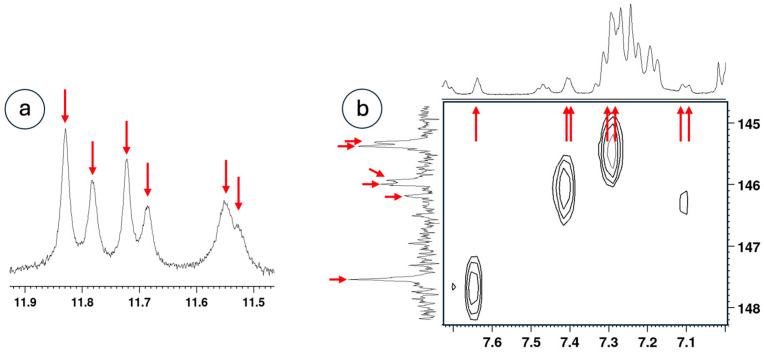
(**a**) The H^+^ region of the 400 MHz ^1^H NMR spectrum of the macrocycle in DMSO-*d_6_* with TFA shows 6 different environments. (**b**) The correlation map from an HSQC experiment showing 7 methine (–NBnN=CH–) resonances, reflecting additional isomers.

Figure 5a shows an expansion of the downfield region of **4**, populated by six broad resonances corresponding to H^+^. Figure 5b shows an expansion of an HSQC experiment showing methine correlations, –NHN=**CH**–CH_2_–. Six different ^13^C methine resonances correlating to seven different methine protons can be identified.

**Figure 6:**
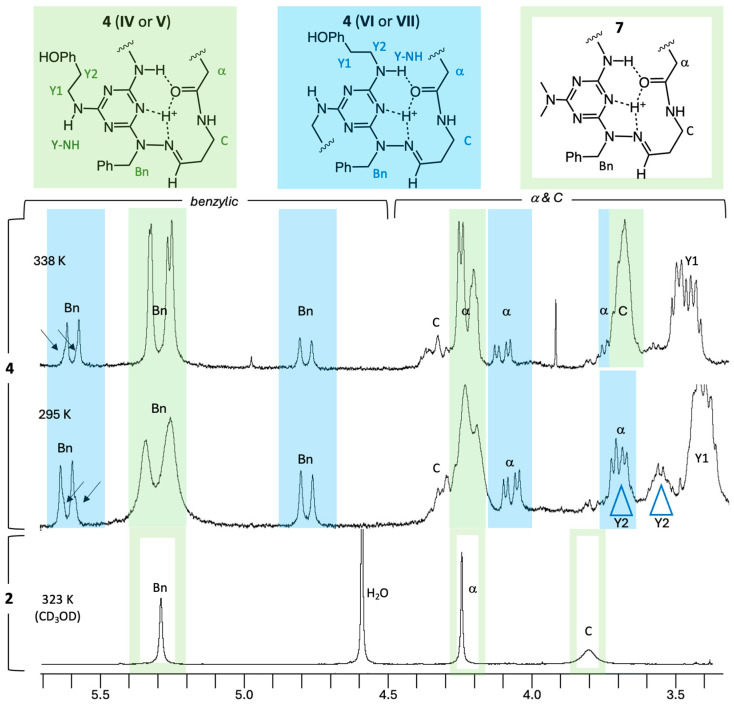
Portions of the 400 MHz ^1^H NMR spectrum of **4** at 295 K and 338 K. Resonances assigned to the traditional conformations (**IV** or **V**) are shown in green boxes. Resonances corresponding to the new conformations (**VI** or **VII**) are shown in blue boxes. Broad peaks sharpen (green; Bn and α) or emerge (green; C) upon heating for the hinge conformations, consistent with the dynamic hinge motion. This behavior is also seen in **2** (taken at 500 MHz in CD_3_OD), which presents a symmetric amine (dimethylamine) leading to a single isomer. Resonances assigned to conformers of **4b** (blue) do not show decoalescence, which is consistent with a static motif. The tyramine group is truncated with “HOPh”. Resonances assigned to multiple conformers (i.e., Y1, Y2) are not assigned a color.

Figure 6 shows an expanded region of the NMR spectra of **4** at room and elevated temperatures. The spectrum of an additional macrocycle, **2** (Figure 2), bearing a symmetric auxiliary amine (dimethylamine) instead of tyramine, is included for comparison.

### 2.4. QTAIM Characterization of the VI–VI Motif

**Figure 7:**
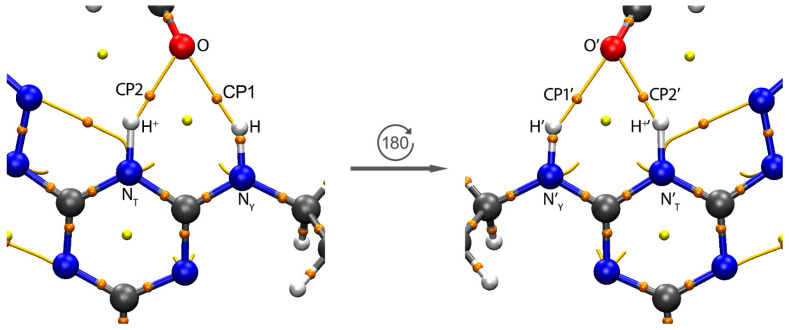
QTAIM molecular graph for the IMHB network in macrocycle **4b**. Bond critical points (type (3, –1)) are depicted as orange spheres, while yellow lines represent the bond paths connecting each hydrogen bond donor and acceptor. The two symmetry-equivalent IHMBs are labeled CP1/CP1′ for N_Y_–H⋯O and CP2/CP2′ for N_T_–H^+^⋯O. The topology confirms that each carbonyl oxygen acts as a dual acceptor, engaging one protonated triazine donor (N_T_–H^+^) and one auxiliary tyramine donor (N_Y_–H⋯O), which is consistent with the **VI**–**VI** motif observed crystallographically. All other atoms of the macrocycle and the non-interacting regions of the molecule are omitted for clarity.

Quantum theory of atoms in molecules (QTAIM) analysis provides a topological description of the electron density that locates bond critical points (CPs) and quantifies the strength and nature of bonding interactions, including hydrogen bonding [25]. To quantify the IMHB network observed crystallographically for **4b**, we carried out a topology analysis of the electron density focused on the two H-bond donors identified in the crystal: the protonated triazine N_T_–H^+^ and the tyramine N_Y_–H. We analyzed the (3, −1) bond critical points (CPs) and bond paths for N_T_–H^+^⋯O (carbonyl) and N_Y_–H⋯O (carbonyl) in both subunits of the macrocycle (**VI**–**VI**) [26]. The BCP parameters are summarized in Table 1, and the corresponding bond paths and CPs are shown in Figure 7. The N_T_–H^+^⋯O contact is stronger than the N_Y_–H⋯O contact, as indicated by a shorter H⋯O distance (1.882 vs. 2.072 Å), higher *ρ*_CP_ (0.195 vs. 0.123 e·Å^−3^), and more stabilizing V_CP_ (−63.8 vs. −35.1 kJ·mol^−1^). Using E_HB_ = 0.5 × *V*_CP_ gives estimated hydrogen bond strengths of −31.9 and −17.6 kJ·mol^−1^, respectively. Both CPs show positive ∇*^2^ρ* and non-negligible values of *ρ* at the CPs, consistent with closed-shell but appreciable H-bonding interactions expected for IMHBs in macrocycles. Equivalent values are observed for the symmetry-related contacts in the second subunit (CP′), consistent with the assigned **VI**–**VI** conformation.

## 3. Discussion

### 3.1. The Introduction of Isomers

**Figure 8:**
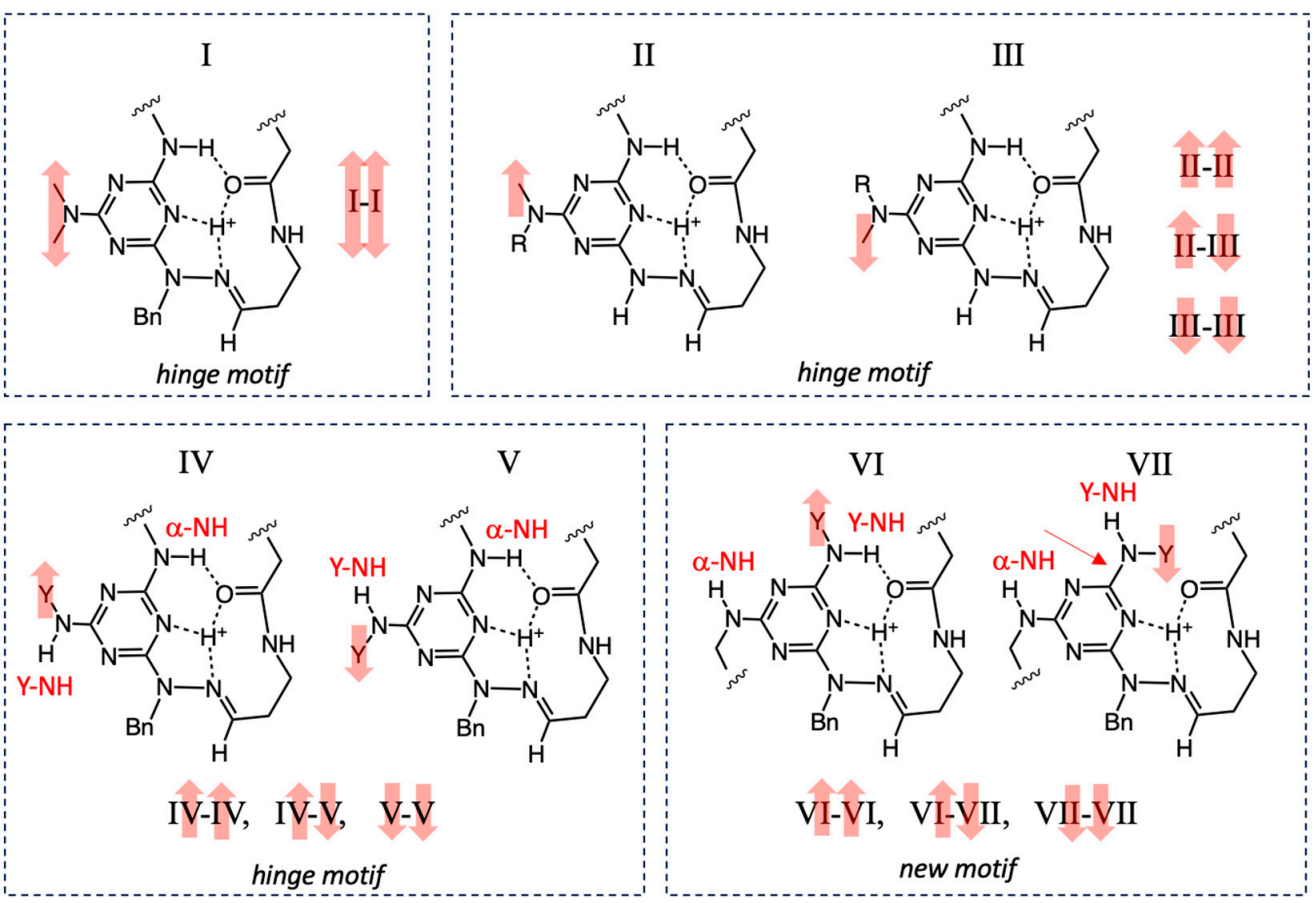
The IMHB networks for auxiliary amines with red arrows to indicate the orientation of reporting methyl groups. Symmetric amines lead to a single conformer (**I**) with a macrocycle, **I-I**, that adopts the hinge motif. Unsymmetric secondary amines that do not provide an alternative IMHB network adopt three conformers resulting from hinge-motif macrocycles adopting **II**, **III**, or both rotamer states. Unsymmetric amines that provide a hydrogen bond donor can have subunits compatible with the hinge motif (**IV** or **V**) or the new motif (**VI** and **VII**). The IMHB network is satisfied for **I** through **VI,** but is unsatisfied for **VII** because the tyramine rotamer arises at the expense of a hydrogen bond.

We define the position where tyramine is attached as the auxiliary position. Substituents here are not immediately attached to the macrocycle backbone. When a symmetric amine is installed at the auxiliary position of a subunit (i.e., dimethylamine, Figure 8, **I**), NMR spectroscopy shows that both subunits of the macrocycle, **I**–**I**, adopt identical conformations leading to a single conformer. The red double arrow shows the orientation of the reporting methyl groups at the auxiliary position.

When an unsymmetric secondary amine is included, the IMHB network is conserved, but hindered rotation about the auxiliary amine position leads to two different environments (**II** and **III**) [24]. These two environments lead to three different conformers in solution, including macrocycles comprising only **II** (**II**–**II**) or only **III** (**III**–**III**), or one containing both **II** and **III** (**II**–**III**). All of these macrocycles adopt the hinge motif.

When tyramine is incorporated, a competing hydrogen bond donor (Y-NH) is introduced, and accordingly, the macrocycle has the opportunity to engage in two different IMHB networks. The IMHB of the hinge motif incorporates α-NH. The new motif incorporates Y-NH into the IMHB.

Access to the original IMHB network and the hinge motif is still available (through **IV** and **V**), with three conformers resulting (macrocycles **IV**–**IV**, **V**–**V**, and **IV**–**V**). When the new IMHB network is accessed (**VI** or **VII**), the hydrogen bond network can be satisfied (**VI**), or the tyramine group can rotate at the expense of a hydrogen bond (**VII**), leading to an unsatisfied IMHB network. The bond rotation that is responsible for the loss of the hydrogen bond is indicated with an arrow on **VII**. Again, three conformers are expected, **VI**–**VI**, **VI**–**VII**, and **VII**–**VII**. In the absence of other benefits or penalties, we would expect the energies of these three isomers to be different, given the cost of zero, one, or two hydrogen bonds for **VI**–**VI**, **VI**–**VII**, or **VII**–**VII**, respectively.

### 3.2. An Existence Theorem—Evidence from X-Ray Crystallography

The X-ray crystal structure shown in Figure 1 establishes that the conformer **VI**–**VI** is accessible. The IMHB network is present and shown with red dotted lines. The carbonyl of one subunit engages in hydrogen bonds with the protonated triazine and the tyramine NH (Y-NH) of the other subunit.

Consistent with the crystal and solution structures of macrocycles adopting conformations **I**–**III**, the p-system of one subunit stacks upon that of the other with triazine rings overlapping with hydrazone groups. Unlike the hinge-motif macrocycles, however, the tyramine groups appear at 180° from each other in an antiperiplanar arrangement. In the hinge motif, the IMHB network orients auxiliary amines at 90°.

QTAIM analysis confirms that the central protonated triazine donor (N_T_–H^+^) preferentially binds the carbonyl oxygen while the N_Y_–H interaction provides the second IMHB in each subunit, reproducing the network observed in the crystal structure. Together, the CP metrics rationalize the static nature of the **VI**–**VI** motif and distinguish it from the hinge network operative in other conformers.

### 3.3. Counting Isomers Theoretically

If conformers (*1*) are persistent on the NMR timescale, (*2*) display chemically distinct environments, and (*3*) are of similar energies, then the number of isomers can be counted using NMR spectra.

For macrocycles that incorporate symmetric auxiliary amines *and provide only one* IMHB (**I**–**I**), symmetry leads to one set of reporting resonances. For example, two methyl resonances are expected because each methyl group is in a unique chemical environment, but both subunits provide identical environments.

For macrocycles that incorporate unsymmetric amines *and provide only one* IMHB network, four reporting resonances (corresponding to three conformers) are expected. Symmetric macrocycles **II**–**II** and **III**–**III** should each produce a single set of reporter resonances due to symmetry. However, **II**–**III** will produce two sets of reporter resonances of equal intensity due to a lack of symmetry across the two subunits [24].

For macrocycles that incorporate unsymmetric amines *and provide two IMHB networks*, six conformers are expected, represented by eight reporting resonances. Single sets of resonances are expected for **IV**–**IV**, **V**–**V**, **VI**–**VI**, and **VII**–**VII**. Two sets of resonances are expected for both **IV**–**V** and **VI**–**VII**. Should differences in the populations of these conformers be reflected by the number of hydrogen bonds, we would expect the resonances for **IV**–**IV**, **V**–**V**, **VI**–**VI**, and **IV**–**V** to be of equal intensity because each has a satisfied IMHB network. A smaller population of **VI**–**VII** would be expected because one hydrogen bond is lost. A still smaller population of **VII**–**VII** would be expected because two hydrogen bonds are lost. The absence of **VII**–**VII** would yield seven reporting resonances.

### 3.4. Counting Isomers Using ^1^H NMR Spectroscopy

Inspection of the 1D ^1^H NMR (Figure 2) confirms that there are more signals than can be attributed solely to **IV**–**IV**, **V**–**V**, and **IV**–**V**, suggesting that additional conformers are accessible in solution (Figure 4).

Initial estimates of the number of isomers derive from the H^+^ region of the spectrum (11.5 ppm–12 ppm), where six resonances are observed (Figure 5a). This region does not provide unequivocal evidence, however, because the resonances are broad and could include the phenolic hydroxyl groups, although we believe they exchange rapidly at room temperature.

Counting the hydrazone methine protons (–NBnN=CH–) in the 1-D ^1^H NMR spectrum has historically yielded accurate isomer counts [4,5,6,7,8,9,10,11]. However, the tyramine and benzyl groups obfuscate this region for **4**. Using an HSQC experiment, both the number of methine hydrogens (–NBnN=CH–) and carbons (–NBnN=CH–) can be counted to provide an estimate (Figure 5b). At least six carbon (–NBnN=CH–) resonances are observed between 145 ppm and 148 ppm. These resonances show correlations to seven different methine hydrogens (–NBnN=CH–). Given the differences in area, we conclude that these conformers are of close, but not identical, energies, and that **VII**–**VII** is likely absent.

### 3.5. Evidence for Different Dynamic Behaviors Differentiates Hinge and New Motifs

The ^1^H NMR spectrum between 3.3 ppm and 5.7 ppm (Figure 4) is especially useful for distinguishing the hinge motif (green) and the new motif (blue).

Previously reported variable temperature NMR of **2** proved remarkably valuable for differentiating between the hinge and new motifs (Figure S8, Supplementary Materials). At low temperature, hinging is slower than the NMR timescale, and the hydrogens of methylene groups (a, C, Bn) are unique, occupying pseudo-axial and pseudo-equatorial positions. Accordingly, they appear as doublets. At higher temperatures, when hinging occurs faster than the NMR timescale, these resonances coalesce and sharpen (Figure 4).

Figure 4 shows that for **4a**, the benzylic resonances observed at 295 K are broad and coalescing. At 338 K, these resonances appear as four well-resolved singlets (corresponding to **IV**–**IV**, **V**–**V**, and **IV**–**V**). The spectra also show a similar evolution of the a-hydrogens. And similar to **2**, wherein the C protons are broadened into the baseline at room temperature, the C protons of **4** are absent at 295 K and emerge as a broad resonance in the spectrum collected at 338 K.

In contrast, multiple doublets are observed for conformers **VI**–**VI**, **VII**–**VII,** and **VI**–**VII**. Both COSY (Figure S8) and HSCQ (Figures S12 and S13, Supplementary Materials) unambiguously identify coupled doublets. In addition, a doublet of Y2 protons of one conformer can also be assigned (blue triangles).

## 4. Materials and Methods

### 4.1. General Methods

NMR spectra were acquired on a 400 MHz Bruker Avance spectrometer. Colorless single crystals of the macrocycle were obtained at ambient temperature via slow evaporation of a water, methanol, and trifluoroacetic acid mixture. X-ray diffraction measurements were performed at room temperature on a Bruker D8 Quest diffractometer equipped with a Photon 100 CMOS detector and Mo Kα radiation (λ = 0.71073 Å). A suitable crystal was mounted on a goniometer head using Paratone-N oil and a MiTeGen MicroLoop LD™, positioned 50 mm from the detector. Data were collected in shutterless mode with an exposure time of 15 s per frame and a scan width of 0.75°, under operating conditions of 50 kV and 30 mA. The Bragg reflections obtained from ω and φ scans were indexed using the APEX3 software (version 2016) suite [27], and data reduction, along with absorption correction, was performed using SAINT [28] and SADABS [29], respectively. The crystal system and space group were identified through XPREP [27] based on Laue symmetry and systematic absences. Structural solution was achieved using the intrinsic phasing method implemented in SHELXT [30], revealing the positions of all non-hydrogen atoms. Subsequent refinements were conducted with the SHELXL [31] program integrated within OLEX2 [32]. Hydrogen atoms were located from the difference Fourier map. During refinement, all non-hydrogen atoms were refined anisotropically, while hydrogen atoms were treated isotropically. Hydrogen atoms bonded to carbon were positioned using the riding model, whereas those attached to heteroatoms were freely refined. Refinements were carried out against *F*^2^ using full-matrix least-squares methods on all observed data. Crystallographic data were deposited with the Cambridge Crystallographic Data Centre as supplementary publication NO. CCDC 2497285.

### 4.2. Synthesis of 5

A solution of 1,1-diethoxypropan-1-amine (2 mmol, 1.0 equiv) in tetrahydrofuran (THF, 20 mL) was stirred with Fmoc-Gly-OSu (2 mmol, 1.0 equiv) at room temperature for 1 h. After completion (TLC monitoring), the mixture was extracted with water and ethyl acetate, and the combined organic layers were dried over anhydrous sodium sulfate and concentrated under reduced pressure. The crude residue was dissolved in THF (16 mL), and diethylamine (2 mL) was added dropwise at room temperature. The mixture was stirred for 3 h, and the solvent was evaporated to afford a crude product used directly in the next step. Cyanuric chloride (2 mmol, 1.0 equiv) was dissolved in THF (10 mL) and cooled to –10 °C. The intermediate from the previous step, dissolved in THF (20 mL) and containing N,N-diisopropylethylamine (DIPEA, 1.4 mL, 8 mmol, 4.0 equiv), was added dropwise to the cold solution and stirred for 20 min at the same temperature. To this mixture, BnHNNHBOC (2.2 mmol, 1.1 equiv) [33] and DIPEA (0.47 mL, 4 mmol, 2.0 equiv) in THF (10 mL) were added, and the reaction was stirred at room temperature for 12 h [19]. The mixture was quenched with brine and extracted with ethyl acetate, and the combined organic layers were dried (Na_2_SO_4_) and concentrated. The residue was purified by silica gel column chromatography using chloroform–methanol (19:1, *v*/*v*) as eluent to afford the desired product as a pure compound as a white solid, 645 mg, 60% yield. ^1^H NMR (400 MHz, DMSO) δ 9.50–8.79 (ms, 1H, NNH), 8.32–7.93 (mt, 1H, NH), 7.93–7.72 (mm, 1H, NH), 7.42–7.18 (m, 5H, Bn), 4.79 (bs, 2H), 4.53–4.39 (m, 1H, A), 3.98–3.69 (md, 2H, a), 3.61–3.47 (m, 2H, OCH_2_), 3.44–3.35 (m, 2H, OCH_2_), 3.14–2.98 (m, 2H, C), 1.69–1.54 (m, 2H, B), 1.46–1.15 (ms, 9H), 1.10 (q, *J* = 7.0 Hz, 6H). ^13^C NMR (100 MHz, CD_3_OD) δ 170.8, 170.6, 170.3, 169.7, 169.1, 167.0, 166.6, 166.1, 156.2, 156.0, 136.5, 136.5, 128.9, 128.2, 128.1, 128.1, 127.3, 127.2, 101.5, 101.3, 80.8, 80.7, 61.7, 61.5, 61.3, 53.5, 53.2, 52.9, 52.8, 44.0, 43.8, 35.1, 35.1, 33.0, 32.9, 27.2, 27.0, 14.3, 14.3, 14.2.

### 4.3. Synthesis of 6

Intermediate **5** (1 mmol, 1 equiv) was dissolved in a 2:1 mixture of tetrahydrofuran (THF, 2 mL) and methanol (1 mL) in a round-bottom flask. DIPEA (2 mmol, 2 equiv) and tyramine (1.1 mmol, 1.1 equiv) were added, and the reaction mixture was stirred at 60 °C for 12 h. Upon completion, water and ethyl acetate were added, and the layers were separated. The organic phase was collected, and the aqueous layer was extracted with ethyl acetate. The combined organic extracts were dried over anhydrous sodium sulfate, filtered, and concentrated under reduced pressure. The residue was purified by silica gel column chromatography using chloroform–methanol (19:1, *v*/*v*) as the eluent to afford the desired product as a white solid (350 mg, 55% yield). ^1^H NMR (400 MHz, DMSO) δ 8.90 (bs, 2H, NH), 8.51 (bs, 3H, NH), 7.44 (t, *J* = 5.4 Hz, 1H, Bn), 7.38–7.20 (m, 4H, Bn), 7.00 (d, *J* = 8.5 Hz, 2H, Y), 6.66 (d, *J* = 8.5 Hz, 2H, Y), 6.57 (bs, 1H, NH), 4.85 (s, 2H, Bn CH_2_), 4.48 (t, *J* = 5.5 Hz, 1H, A), 3.82 (d, *J* = 6.0 Hz, 2H, a), 3.60–3.50 (m, 2H, OCH_2_), 3.47–3.39 (m, 2H, OCH_2_), 3.37 (t, *J* = 7.7 Hz, 2H,Y1), 3.13 (t, *J* = 6.6 Hz, 2H, C), 2.70 (t, *J* = 7.7 Hz, 2H, Y2), 1.67 (q, *J* = 6.6 Hz, 2H, B), 1.35 (s, 9H, BOC), 1.11 (t, *J* = 7.0 Hz, 6H, CH_3_). ^13^C NMR (100 MHz, CD_3_OD) δ 171.8, 167.0, 166.1, 155.4, 137.9, 130.3, 129.5, 128.0, 126.9, 114.8, 101.6, 80.4, 61.6, 52.6, 44.1, 42.4, 35.0, 34.8, 32.9, 27.3, 14.3.

### 4.4. Synthesis of the Macrocycle

TFA (1.0 mL) was added to a stirred solution of **6** (0.020 g, 0.03 mmol) in CH_2_Cl_2_ (1.0 mL). The mixture was stirred for 12 h at room temperature. The solvent was removed by rotary evaporation under reduced pressure and dried to afford the macrocycle as a white powder.

### 4.5. QTAIM Calculations

The molecular geometry was extracted from the single-crystal X-ray structure of **4b**. Because X-ray hydrogen positions are less reliable than heavy atoms, and our objective was to characterize the solid-state H-bond topology without relaxing the solid-state scaffold, we only optimized hydrogen atoms while constraining all non-hydrogen atoms to their experimental coordinates. This constrained refinement yields realistic X–H distances and orientations while preserving the crystal-observed conformation. The macrocycle was modeled in its crystallographically consistent dicationic state (+2). The H-only constrained optimization was performed at wB97X-D/cc-pVDZ in the gas phase, and a single-point calculation at wB97X-D/aug-cc-pVTZ provided the electron density for QTAIM using the Gaussian 16 suite of programs [34]. QTAIM analyses were performed with Multiwfn [35] on the single-point density.

## 5. Conclusions

This work explores the impact of an additional IMHB network on the distribution of conformers (and their motifs) for 24-atom triazine macrocycles. When a single IMHB network is available, the resulting macrocycles adopt a common hinge motif and engage in hinge motion.

Introducing a primary amine at the auxiliary position provides an additional hydrogen bond donor and introduces a new IMHB network. This network allows access to a new motif. The observation of multiple conformers in solution by ^1^H NMR spectroscopy corroborates this hypothesis. The crystal structure provides evidence of one structure that adopts the new IMHB network, orienting the auxiliary tyramines at 180° to each other.

The hydrogen bonds that result can be described using QTAIM. The interaction between the carbonyl and Y-NH can be described as a moderately strong hydrogen bond that is electrostatic in nature and directional, possessing significant s-character. The interaction between the protonated triazine and the carbonyl shows similar characteristics but is stronger than the aforementioned interaction.

While conformations in solution and the solid state can be markedly different, we are inclined to believe that the crystal structure of the new motif corresponds to the solution structure observed. Evidence from 1-D ^1^H NMR spectroscopy is limited to the dramatic downfield shift in the tyramine-NH groups, Y-NH, consistent with hydrogen bonding. Regrettably, two-dimensional NMR experiments (ROESY and NOESY) do not yield definitive corroboration due to spectral crowding. Additional macrocycles are currently being prepared to probe the generality of these observations. More tenuous support comes from the knowledge that the solution and solid-state structures of macrocycles adopting the hinge motif are well correlated. And here, the hinge motif conformers can be identified due to the behaviors observed in the variable temperature ^1^H NMR spectra.

In contrast, the sharpness of many of the resonances in the ^1^H NMR spectrum assigned to the new motif across a broad temperature range (room temperature to 65 °C) suggests that it is relatively static without appreciable hinging motion under ambient conditions.

The emergence of new, persistent macrocycle conformers is significant when considering applications in either materials science or medicine. When it comes to designing macrocyclic drugs, having access to diverse, persistent conformations facilitates design. For polymer design, the physical properties of materials derived from these conformers, **4a** or **4b,** would be expected to be very different, given that one conformer engages readily in hinge motion and the other appears resistant.

## Figures and Tables

**Table 1:** QTAIM parameters at (3, −1) critical points (CPs) for intramolecular H-bonds in **4b** (**VI**–**VI**) at ωB97XD/AUG-cc-pVTZ level of DFT calculations (D_A-H_ = O⋯H distance, D_D-H_ = N–H distance, D_A-CP_ = O⋯CP distance, D_H-CP_ = H⋯CP distance, ρ_CP_ = electron density at CP, ∇^2^ρ_CP_ = Laplacian of ρ, ε = bond ellipticity, G_CP_ = bond kinetic energy density, V_CP_ = bond potential energy density, and E_HB_ = hydrogen-bond energy).

Property	CP1	CP2	CP1′	CP2′
Interaction	N_Y_–H⋯O	N_T_–H^+^⋯O	N′_Y_–H′⋯O′	N′_T_–H′^+^⋯O′
D_A-H_ (Å) ^a^	2.072	1.882	2.073	1.882
*D*_D-H_ (Å) ^b^	1.019	1.031	1.019	1.031
*D*_A-CP_ (Å) ^c^	1.298	1.216	1.298	1.216
*D*_H-CP_ (Å) ^d^	0.779	0.667	0.779	0.667
ρ_CP_ (e·Å^−3^) ^e^	0.123	0.195	0.123	0.195
∇^2^*ρ*_CP_ (e·Å^−5^) ^f^	1.79	2.39	1.79	2.39
ε ^g^	0.040	0.088	0.040	0.088
*G_CP_* (kJ·mol^−1^) ^h^	41.8	64.5	41.8	64.5
*V_CP_* (kJ·mol^−1^) ^i^	−35.1	−63.8	−35.1	−63.9
*E_HB_* (kJ·mol^−1^) ^j^	−17.6	−31.9	−17.6	−31.9

^a^ Shorter acceptor distances are consistent with a stronger hydrogen bond. ^b^ Values consistent with N-H covalent bonds. ^c^ The BCP lies closer to O in both cases. ^d^ CP2 shifts towards hydrogen, reflecting a stronger and more polarized hydrogen bond. ^e^ The values reflect intermediate hydrogen bond (0.04–0.20), although CP2 borders on the strong limit. ^f^ Electrostatics dominate with high charge depletion between H and O and accumulation near nuclei. ^g^ Cylindrical (s-like) density for CP1; similar but possible p-character or polarization to O. ^h^ Larger charge polarization at the CP2, which is consistent with a more electrostatic interaction. ^i^ Stronger stabilizing interactions at CP2 and greater covalent character of the hydrogen bond. ^j^ CP1 and CP2 are moderate and strong hydrogen bonds, respectively, with the latter bordering on ionic.

## Data Availability

All data is available in the Supplementary Materials.

## Supplementary Materials

The following Supplementary Materials can be downloaded at https://www.mdpi.com/article/10.3390/molecules30224475/s1; Figure S1. Conformer Chart, Table S1. Crystallographic and refinement statistics, Table S2. Hydrogen-bond geometry (Å, º), Figure S2. The 400 MHz ^1^H NMR spectrum of **5** in DMSO-*d_6_*, Figure S3. The 100 MHz ^13^C NMR spectrum of **5** in DMSO-*d_6_*, Figure S4. The 400 MHz 1H NMR spectrum of **6** (monomer) in DMSO-*d_6_* at 295 K, Figure S5. The 400 MHz ^1^H NMR spectrum of **6** (monomer) in DMSO-*d_6_* at 338 K, Figure S6. The 100 MHz ^13^C NMR spectrum of **6** (monomer) in DMSO-*d_6_* at 295 K, Figure S7. Low and high temperature ^1^H NMR spectra of **4** in DMSO-*d_6_* at 400 MHz, Figure S8. An expanded region of the 400 MHz COSY NMR spectrum of **4** in DMSO-*d_6_*, Figure S9. Variable temperature ^1^H NMR spectroscopy distinguishes motifs, Figure S10. Downfield regions of 100 MHz 1-D ^13^C and DEPT135 spectra in DMSO-*d_6_* at 295 K, Figure S11. The entire DEPT135 spectrum with insets. Figure S12. HSQC experiment of **4** in DMSO-*d_6_*, Figure S13. Expanded region of the HSQC experiment of **4** in DMSO-*d_6_.* Figure S14. An LCMS trace of **4,** suggesting both motifs.

## Abbreviations

The following abbreviations are used in this manuscript:

BOC	*tert*-butyloxycarbonyl
CP	Critical point
DIPEA	Diisopropylethylamine
Fmoc	9-fluorenylmethoxycarbonyl
Gly-OSu	Glycine succinimide ester
IMHB	Intramolecular Hydrogen Bond
QTAIM	Quantum Theory of Atoms in Molecules
TLC	Thin-layer chromatography
THF	Tetrahydrofuran
