# Supplementary material for: A Competing Hydrogen Bond Network Offers Access to a New Conformation in 24-Atom Triazine Macrocycles
Source: Molecules. 2025 Nov 20;30(22):4475. doi: 10.3390/molecules30224475 (PMC12655256; doi:10.3390/molecules30224475)

SUPPORTING INFORMATION

A Competing Hydrogen Bond Network Offers Access to a New Conformation in 24-atom Triazine Macrocycles

K. Harsha Vardan Reddy [1], Arshad Mehmood [2], Akop Yepremyan [1] and Eric E. Simanek [1,*]

[1] Department of Chemistry & Biochemistry, Texas Christian University, Fort Worth, TX 76129, USA; h.r.kasireddy@tcu.edu (K.H.V.R.)
[2] Institute for Advanced Computational Science, Stony Brook University, Stony Brook, NY 11794, USA
* Correspondence: e.simanek@tcu.edu

TABLE OF CONTENTS

**Figure S1. Conformer Chart**	S2
**Table S1. Crystallographic and refinement statistics.**	S3
**Table S2. Hydrogen-bond geometry (Å, º)**	S3
**Figure S2. The 400 MHz ^{1}H NMR spectrum of 5 in DMSO-d_6**	S4
**Figure S3. The 100 MHz ^{13}C NMR spectrum of 5 in in DMSO-d_6**	S5
**Figure S4. The 400 MHz ^{1}H NMR spectrum of 6 (monomer) in DMSO-d_6 at 295 K**	S6
**Figure S5. The 400 MHz ^{1}H NMR spectrum of 6 (monomer) in DMSO-d_6 at 338 K**	S7
**Figure S6. The 100 MHz ^{13}C NMR spectrum of 6 (monomer) in DMSO-d_6 at 295 K**	S8
**Figure S7. Low and high temperature spectra of 4 in DMSO-d_6 at 400 MHz**	S9
**Figure S8. An expanded region of the 400 MHz COSY NMR spectrum of 4 in DMSO-d_6**	S10
**Figure S9. Variable temperature ^{1}H NMR spectroscopy distinguishes conformers**	S11
**Figure S10. Downfield regions of 100 MHz 1-D ^{13}C and DEPT135 spectra in DMSO-d_6 at 295 K**	S12
**Figure S11. The entire DEPT135 spectrum with insets**	S13
**Figure S12. HSQC experiment of 4 in DMSO-d_6**	S14
**Figure S13. Expanded region of the HSQC experiment of 4 in DMSO-d_6**	S15
**Figure S14. An LCMS trace suggesting both motifs**	S16

Figure S1. Conformer Chart

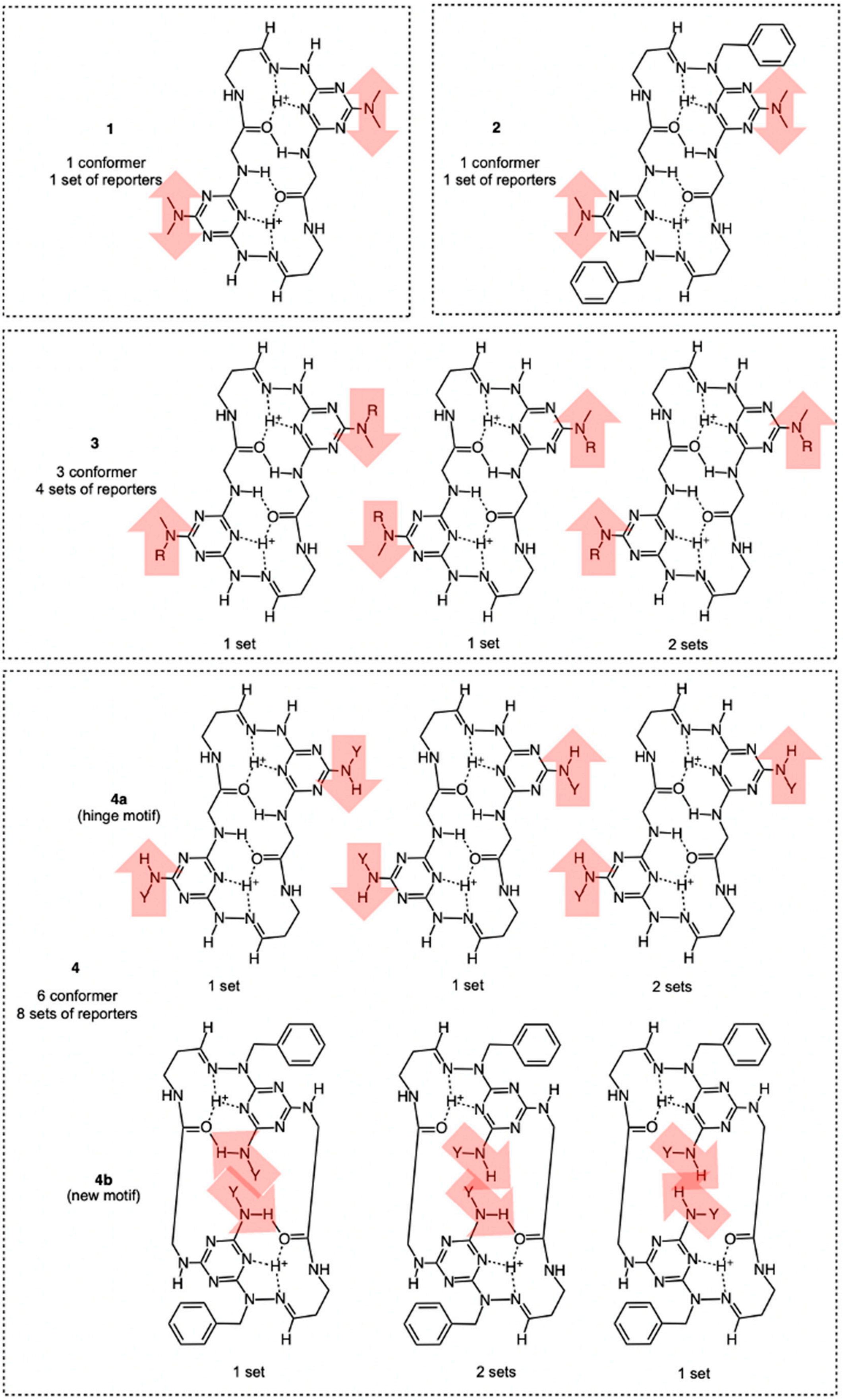

Table S1. Crystallographic and refinement statistics.

**Crystal data**	
Chemical formula	$C_{46}H_{54}N_{16}O_4 \cdot 2(C_2F_3O_2)$
M_r	1121.09
Crystal system, space group	Triclinic, *P*-1
Temperature (K)	100(1)
a, *b*, *c* (Å)	9.3220 (8), 11.7758 (9), 13.0947 (10)
α, β, γ (°)	101.487 (2), 108.494 (2), 105.473 (2)
V (Å^3)	1248.89 (17)
Z	1
Radiation type	Mo $K\alpha$ (λ = 0.71073 Å)
μ (mm^{-1})	0.12
Crystal size (mm)	0.40 × 0.20 × 0.19
Crystal shape and color	Block, colourless
**Data collection**	
Diffractometer	Bruker D8 Quest
Absorption correction	Multi-scan
T_{min}, T_{max}	0.700, 0.745 [$I > 2\sigma(I)$]
No. of measured, independent and observed reflections	27424, 3060, 2690
R_{int}	0.031
θ (°)	2.9–22.0
$(\sin \theta/\lambda)_{max}$ (Å^{-1})	0.527
**Refinement**	
$R[F^2 > 2\sigma(F^2)]$, $wR(F^2)$, S	0.043, 0.118, 1.08
No. of reflections	3060
No. of parameters	362
$\Delta\rho_{max}$, $\Delta\rho_{min}$ (e Å^{-3})	0.40, -0.34

Table S2. Hydrogen-bond geometry (Å, º)

D—H···*A*	*D*—H	H···*A*	*D*···*A*	*D*—H···*A*
O2—H2···O3	0.84	1.76	2.603 (3)	176
N1—H1···O1	0.88	2.05	2.782 (3)	140
N5—H5···O2^{i}	0.88	2.08	2.897 (3)	154
N4—H4···O1	0.88	2.27	2.949 (3)	133
N8—H8···O4^{ii}	0.88	2.01	2.863 (3)	164

Symmetry codes: (i) *x*, *y*+1, *z*; (ii) *x*, *y*+1, *z*+1; (iii) *x*+1, *y*+1, *z*+1.

Figure S2. The 400 MHz ^{1}H NMR spectrum of 5 in DMSO-*d*$_{6}$. Multiple rotamers are evident.

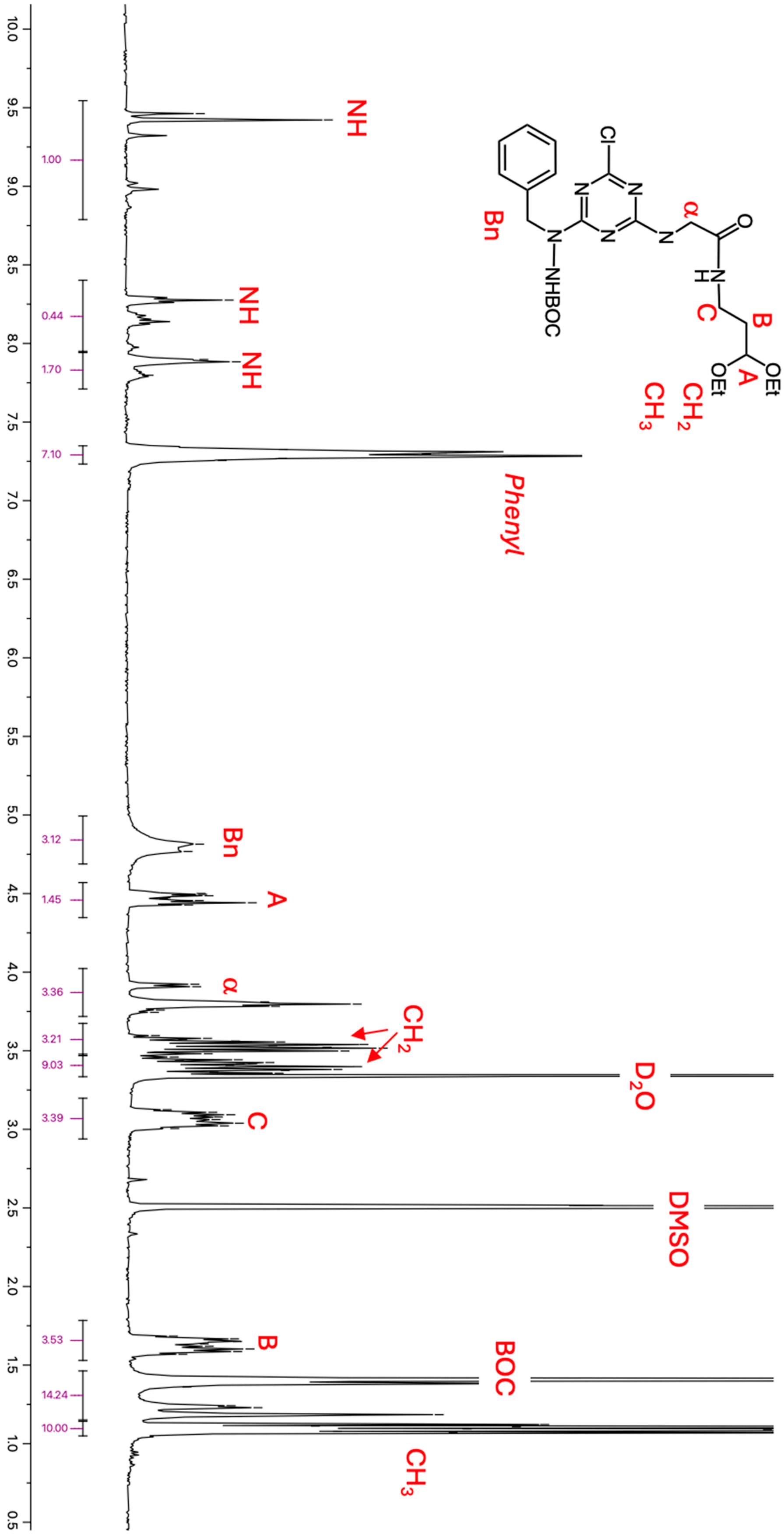

Figure S3. The 100 MHz ^{13}C NMR spectrum of 5 in in DMSO-*d*$_6$. Multiple rotamers are evident.

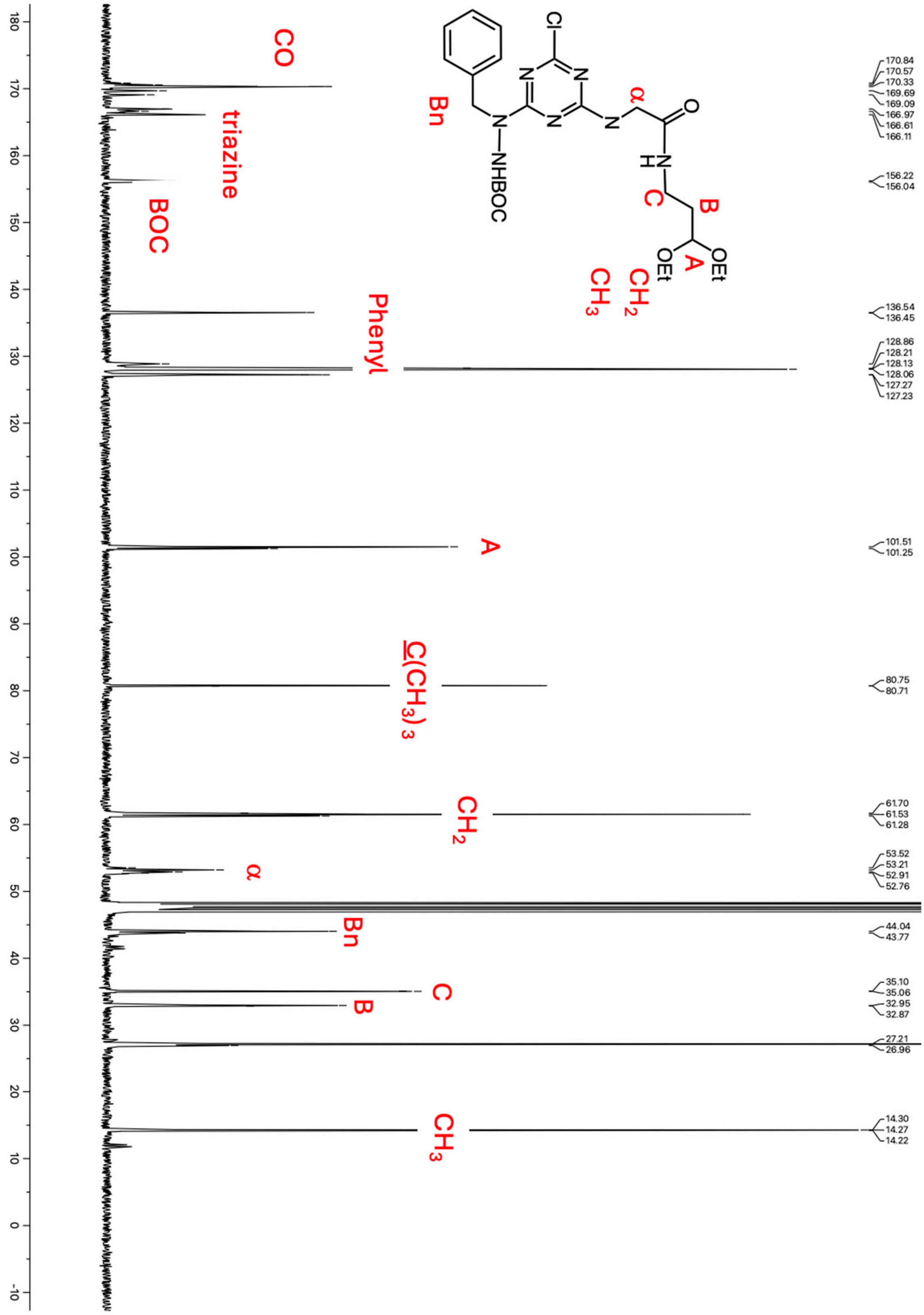

Figure S4. The 400 MHz ^{1}H NMR spectrum of 6 (monomer) in DMSO-d_6 at 295 K. Rotamers give rise to broad resonances.

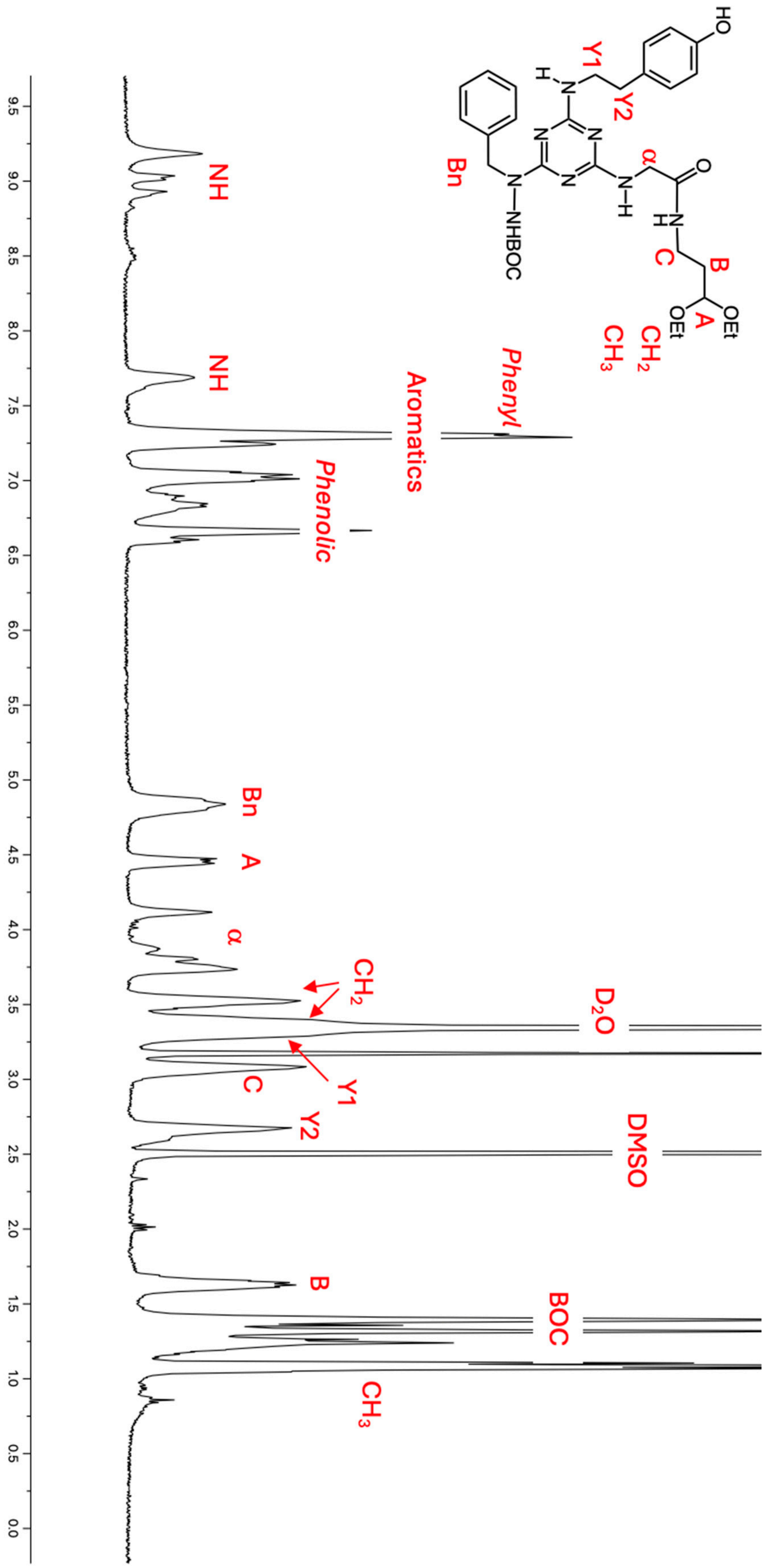

Figure S5. The 400 MHz ^{1}H NMR spectrum of 6 (monomer) in DMSO-d_6 at 338 K. At elevated temperature a single set of resonances are observed for a rapidly equilibrating rotamer population.

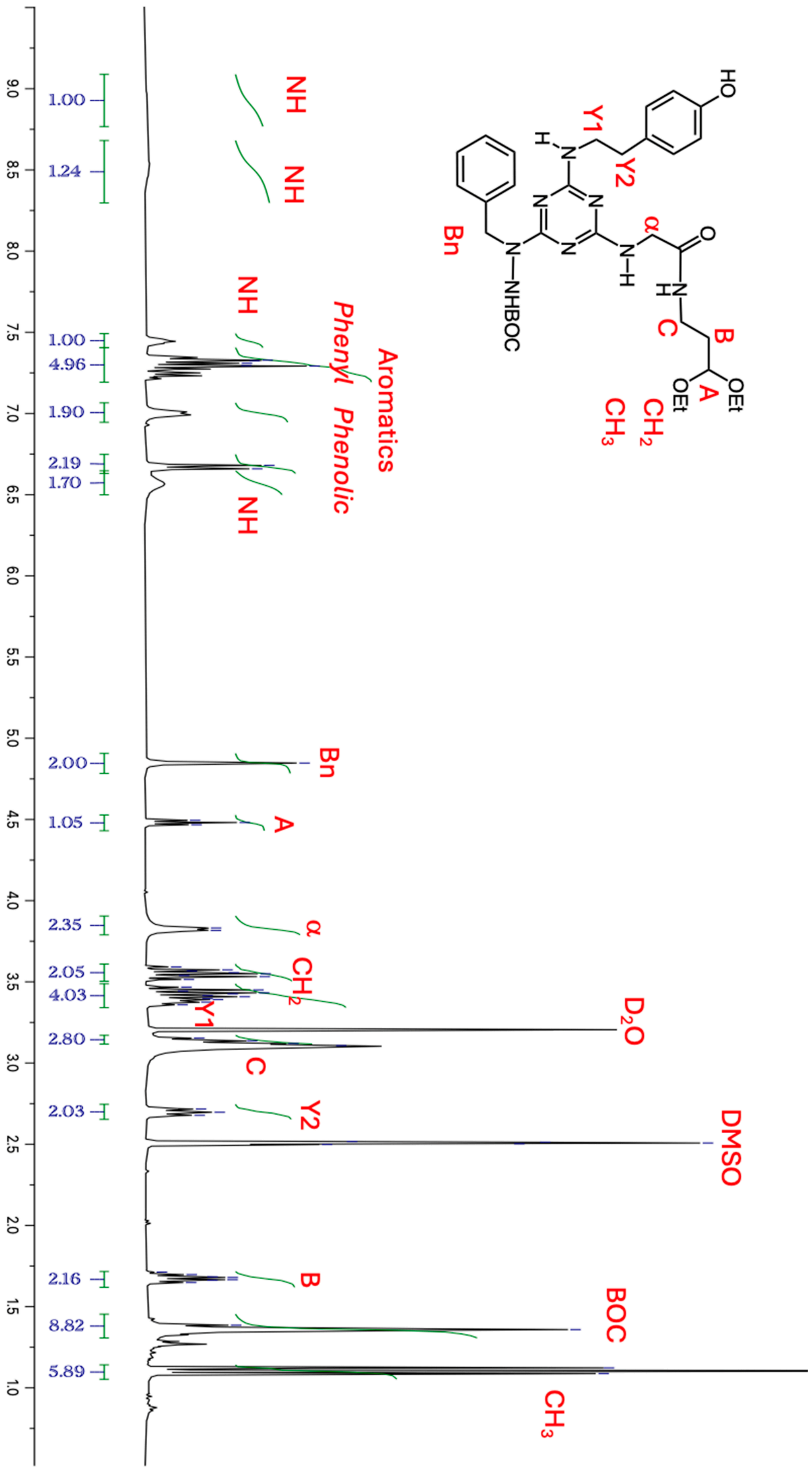

Figure S6. The 100 MHz ^{13}C NMR spectrum of 6 (monomer) in DMSO-d_6 at 295 K. The ^{13}C NMR of spectrum of **1** appears as beneath to guide the eye.

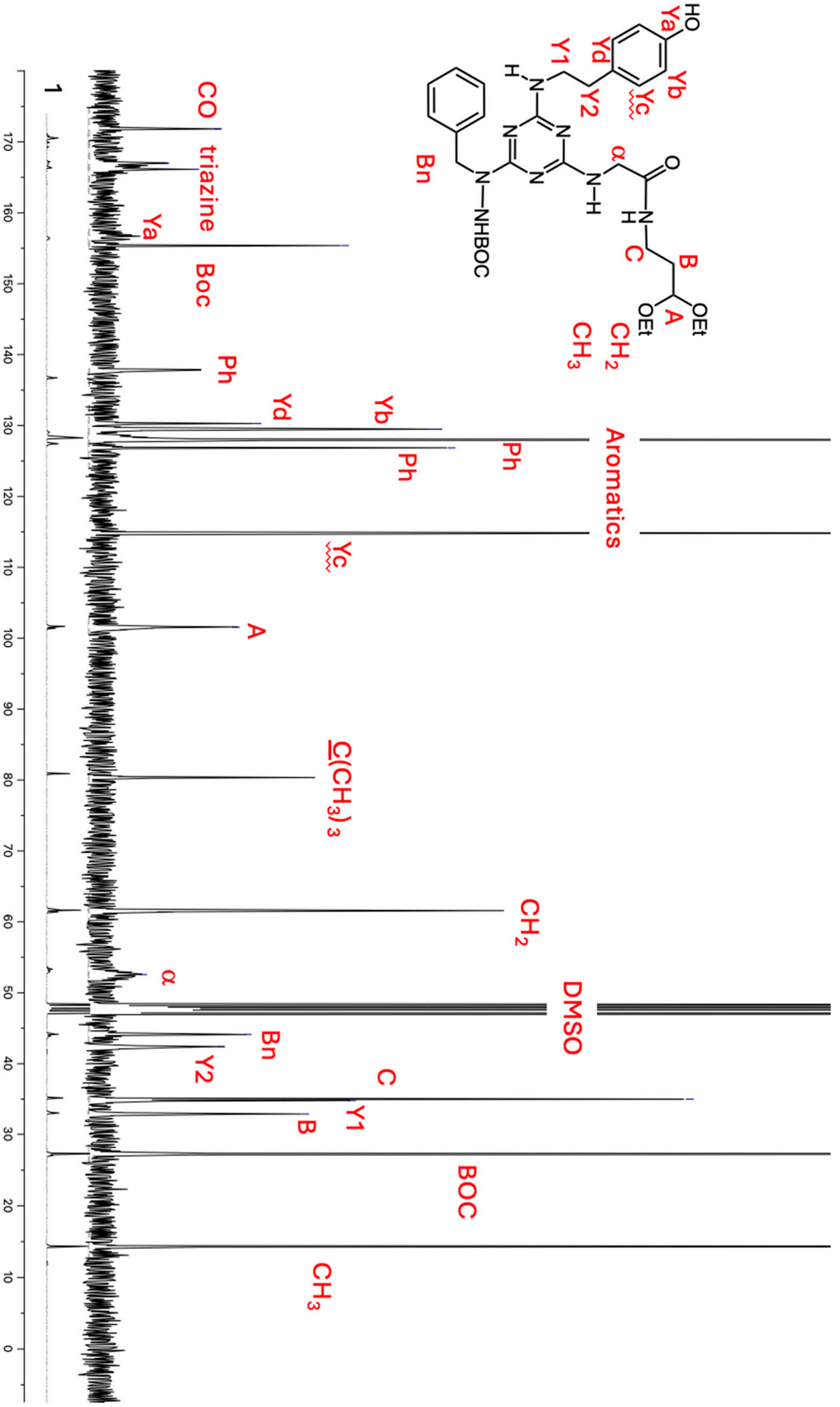

Figure S7. Low and high temperature spectra of 4 acquired in DMSO-d_6 at 400 MHz. The dynamic (hinge-like) motion of **V** and **VI** (red labels) is revealed with the sharpening of the benzylic (Bn) and α resonances. Resonances corresponding to C are broadened into the baseline at 295 K, but emerge as a coalesced peak at 338 K. The persistence of the new conformer, **IV**, is reflected in the well-resolved resonances (blue labels) that are smaller and are diastereotopic with each hydrogen of methylenes Bn, α, and Y1 resolved due to unique chemical environments and exchange (if any) slower than the NMR timescale.

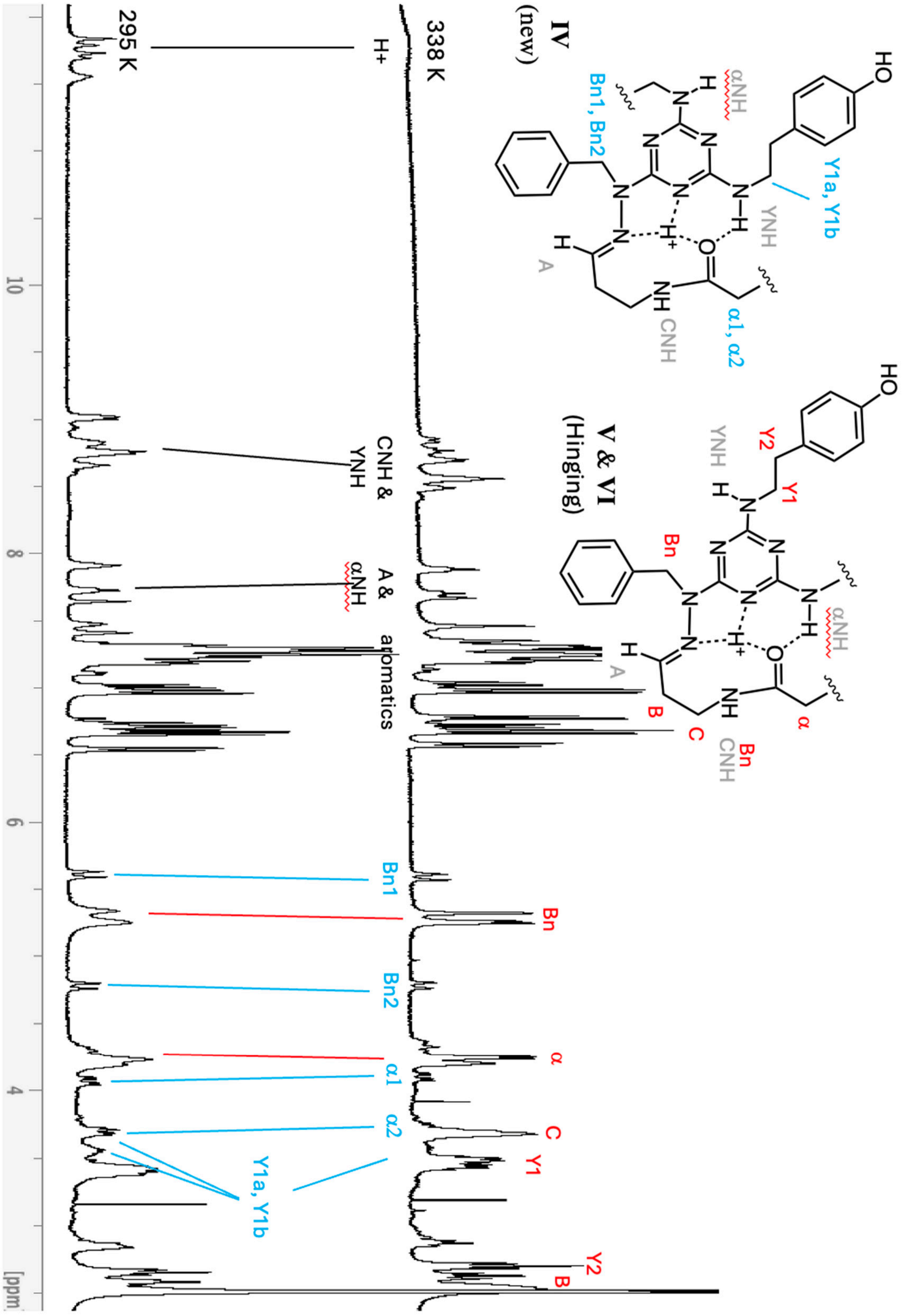

Figure S8. An expanded region of the 400 MHz COSY NMR spectrum of 4 in DMSO-d_6. The spectrum shows correlations for conformation **IV** in red. Two isomers are readily distinguished by the benzylic resonances and identified as “major” and “minor”.

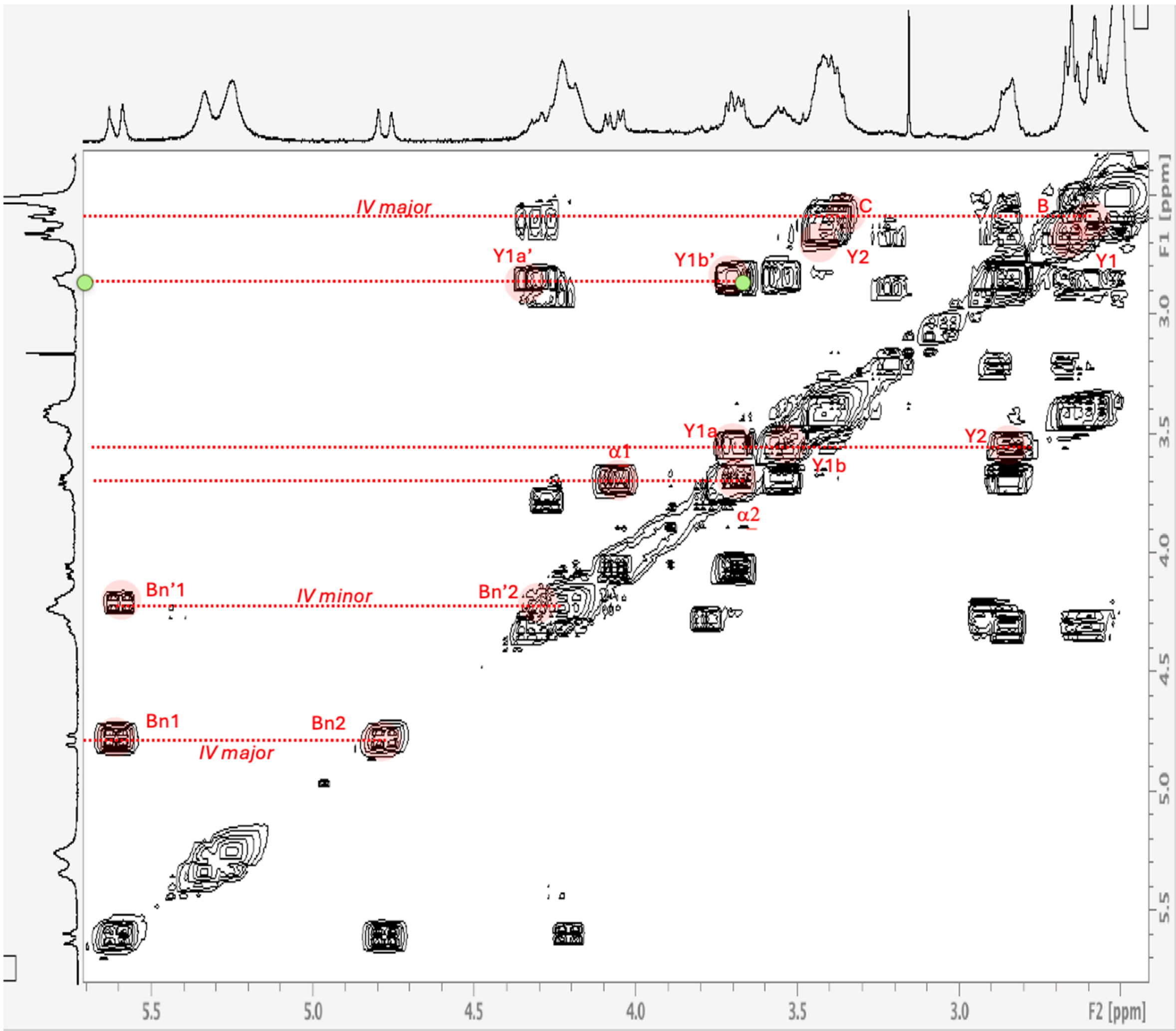

Figure S9. Variable temperature ^{1}H NMR spectroscopy distinguishes conformers. Variable temperature ^{1}H NMR spectra at 500 MHz in CD_3CN of a previously described macrocycle (inset) lacking tyramine (bottom) and the spectra of the macrocycle at 21 ºC and 65 ºC. The colors indicate complementary resonances of both molecules. Benzylic (green) and α (blue) resonances are coalesced at 21 ºC for the macrocycle lacking tyramine (bottom), but the C hydrogens (red) are broadened into the baseline. Similar behavior is observed for the macrocycle reported here (top) acquired at 400 MHz in DMSO-d_6. The data supports the hypothesis that differences in dynamic behavior allows for conformer **IV** to be distinguished from **V** and **VI**. Stars indicate the diastereotopic methylenes attributed to the new conformer, **IV**.

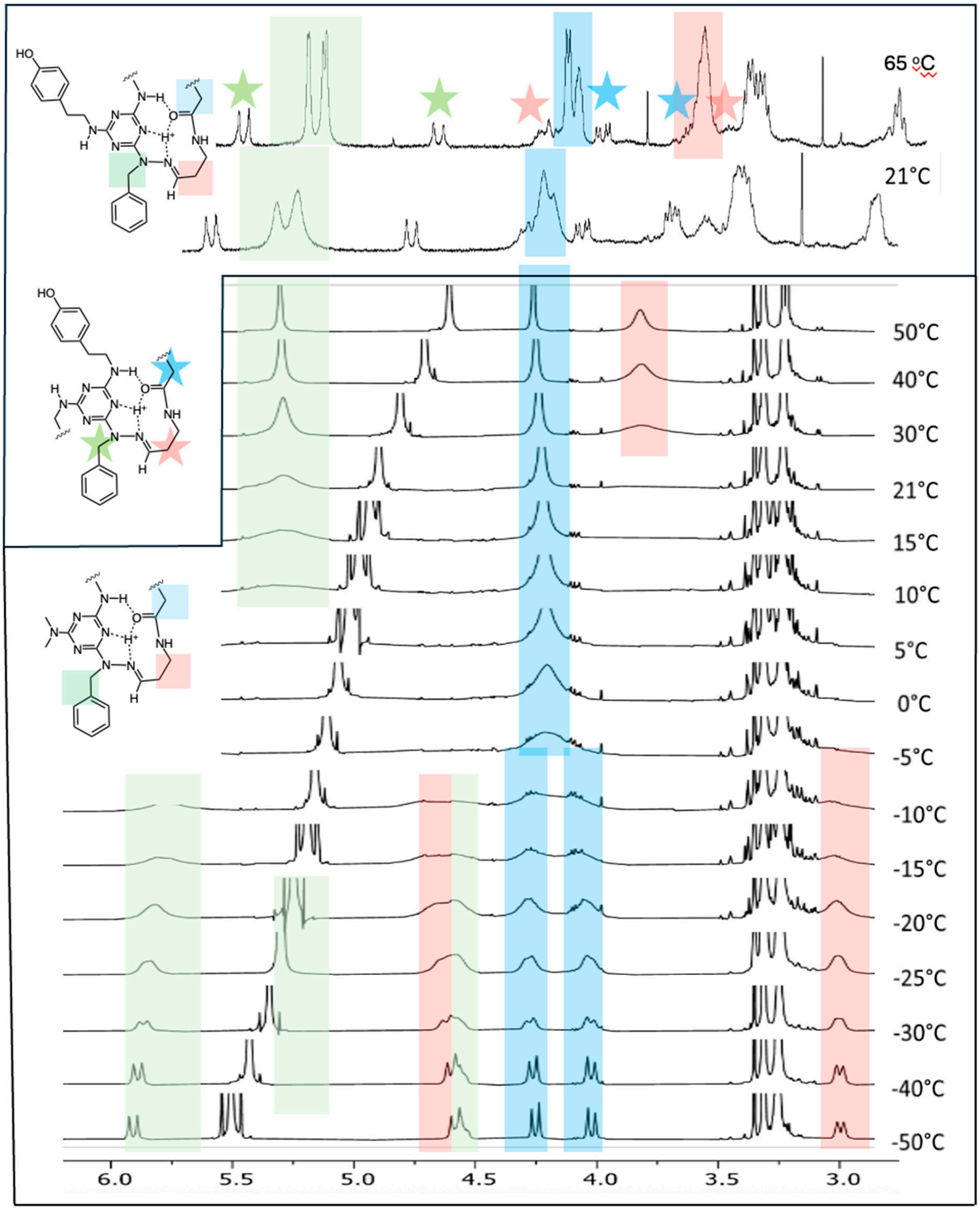

Figure S10. Downfield regions of 100 MHz 1-D ^{13}C and DEPT135 spectra acquired from DMSO-d_6 at 295 K. Both spectra show an abundance of isomers. The inset shows resonances corresponding to A for the multiple conformers. Six unique environments are observed.

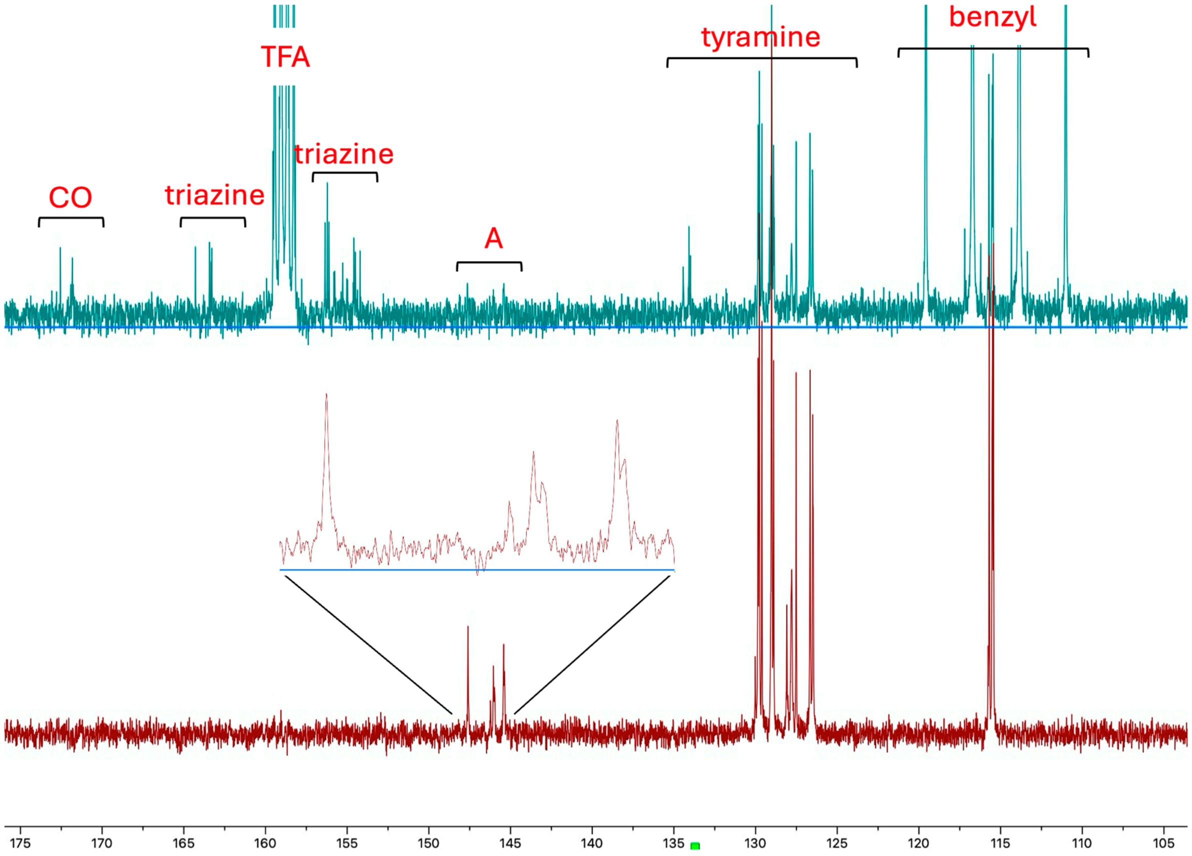

Figure S11. The entire DEPT135 spectrum with insets. Inset (a) shows the entire spectrum acquired for the macrocycle at 295 K in DMSO-d_6. Insets show the A region (b), a portion of the phenolic region (c), the aromatic benzyl and tyramine regions (d) and the upfield region (e).

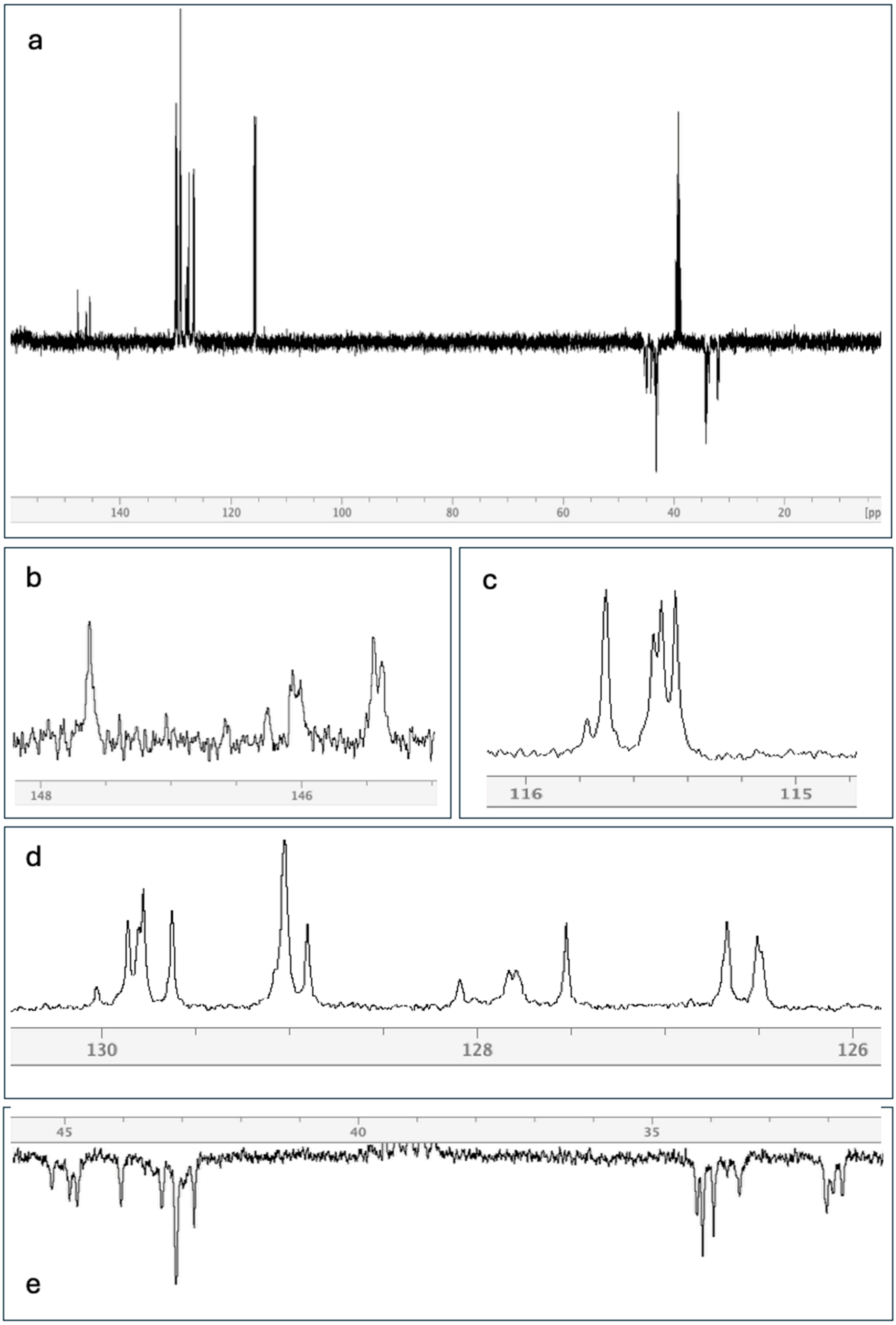

Figure S12. HSQC experiment of 4 in DMSO-*d*$_6$. The spectrum experiment was performed at 295 K on a 400 mHz NMR Bruker Avance spectrometer.

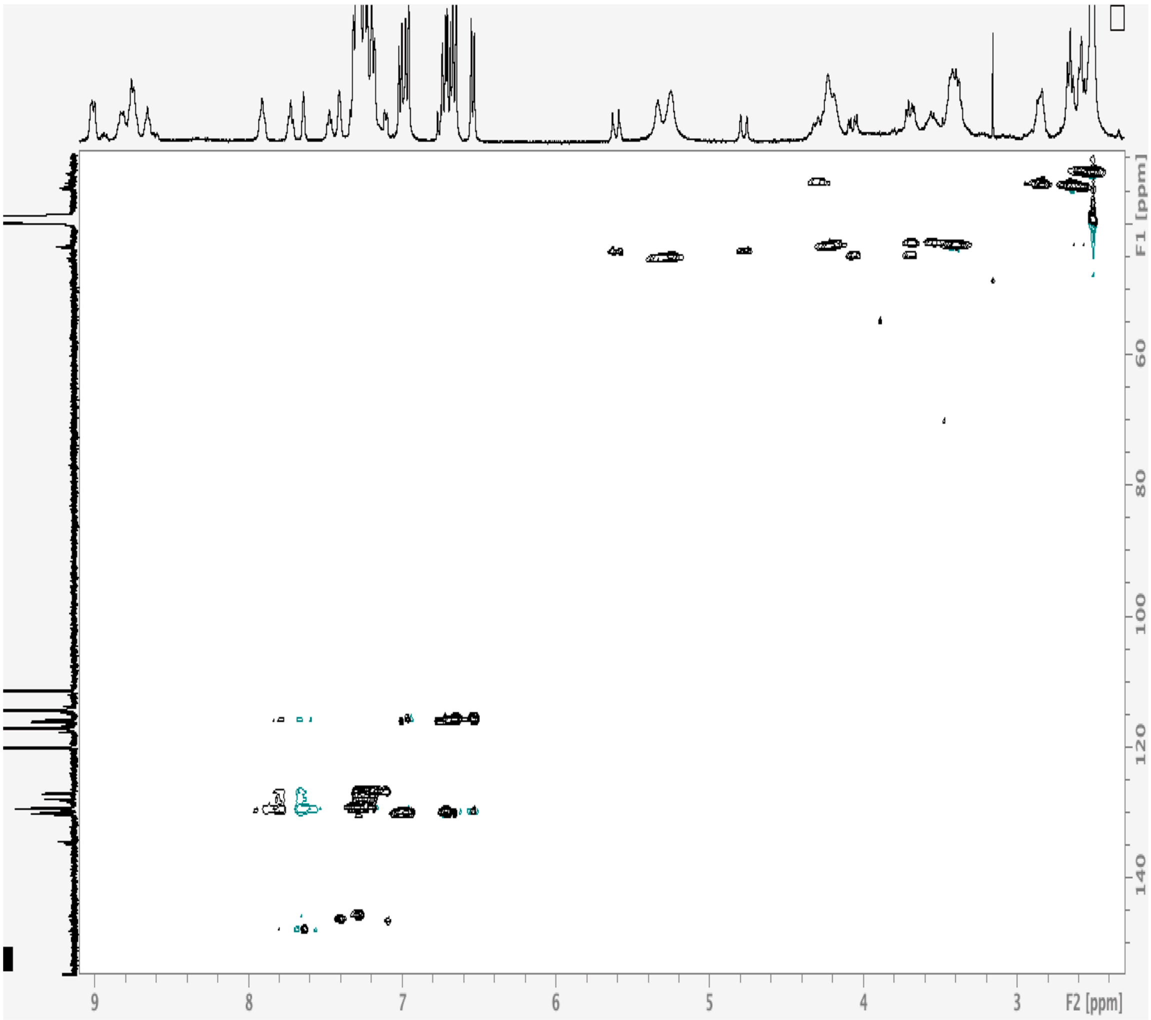

Figure S13. Expanded region of the HSQC experiment of 4 in DMSO-*d*$_6$. The data was acquired on a 400 mHz NMR Bruker Avance spectrometer. The ^{13}C spectrum displayed on the Y-axis is derived from a separate HSQC experiment, chosen for far better resolution. The correlations confirm assignment of diastereotopic methylene hydrogens.

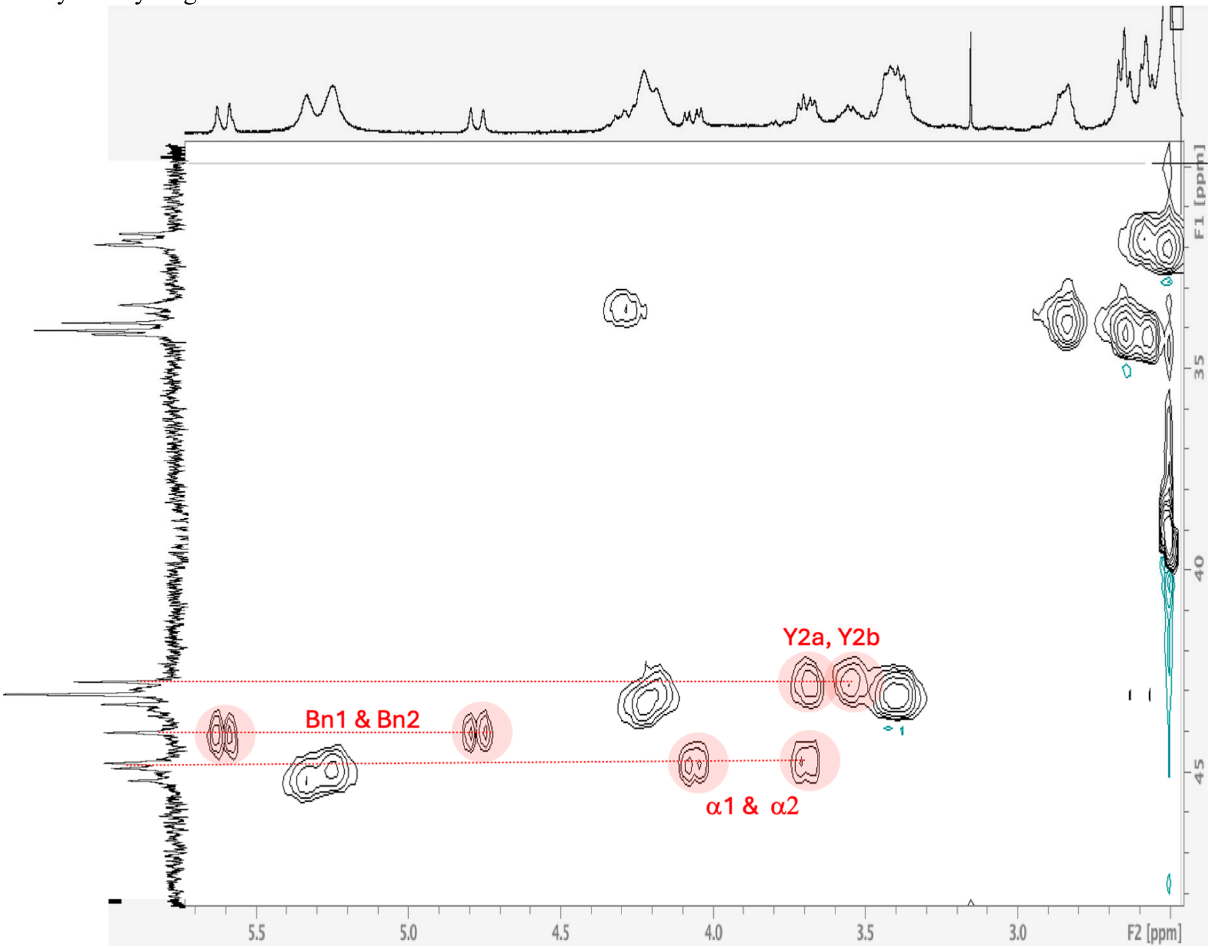

Figure S14. An LCMS trace suggesting both motifs. Collected at the Shimadzu Center at the University of Texas in Arlington, the LCMS shows two peaks eluting from the column. Each peak shows a molecular ion at an *m/z* expected for **4** leading us to the hypothesis that one corresponds to **4a** and the other to **4b**.

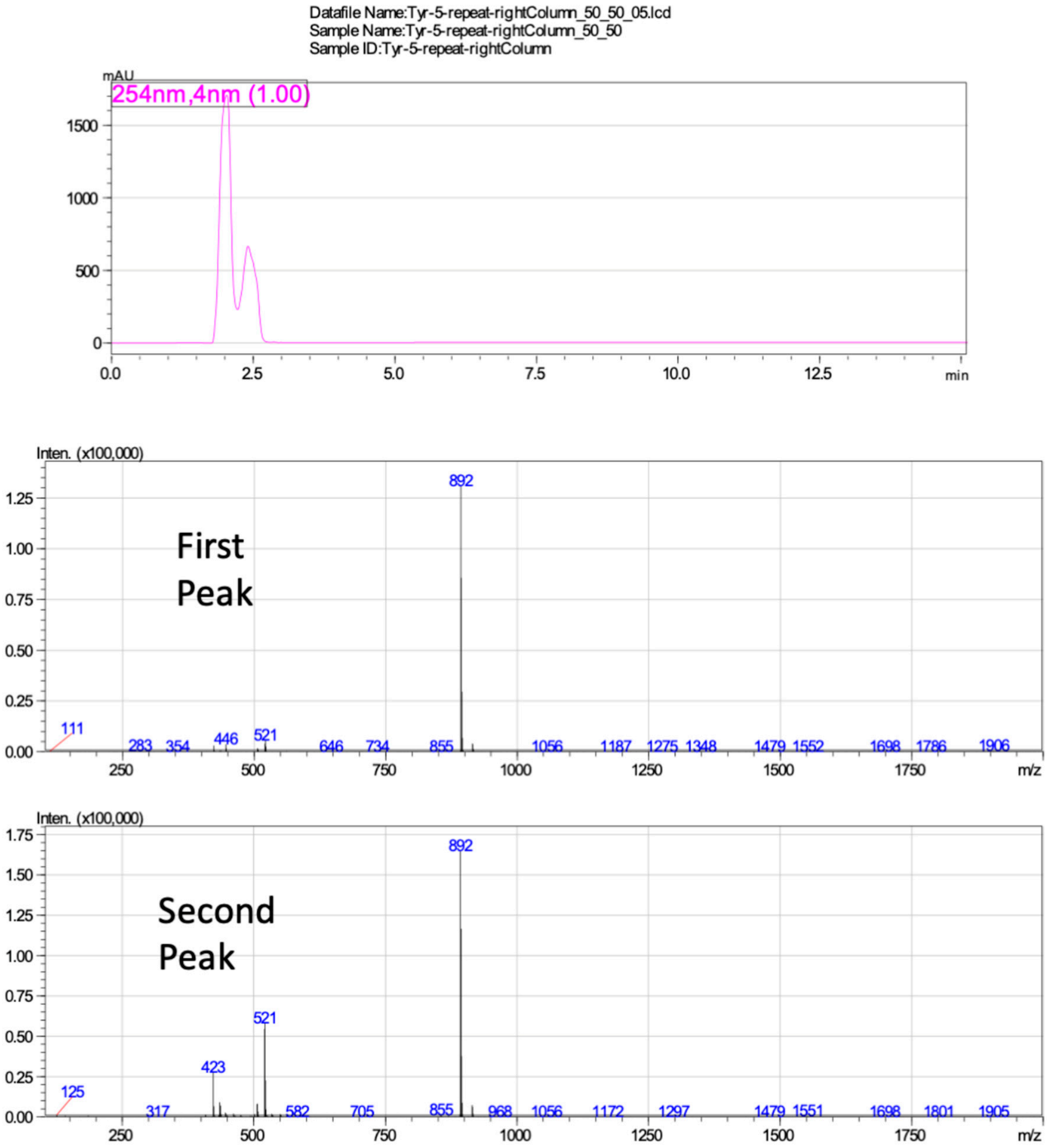

Supplement: Supplementary file 1 [file molecules-30-04475-s001.zip › molecules-3968436-supplementary.pdf]
